# Diminuendo al bottom—Clarifying the semantics of music notation by re-modeling

**DOI:** 10.1371/journal.pone.0224688

**Published:** 2019-11-22

**Authors:** Markus Lepper, Michael Oehler, Hartmuth Kinzler, Baltasar Trancón y Widemann

**Affiliations:** 1 Semantics GmbH, Berlin, Germany; 2 Osnabrück University – Institute for Musicology and Music Pedagogy, Osnabrück, Germany; Instituto Nacional de Medicina Genomica, MEXICO

## Abstract

One of many aspects of musical notation is that of a graphical language which strives to be totally precise, but falls short because it has been defined by historical evolution, cultural construction and de-central ramification. This article applies standard techniques for computer languages to reconstruct a precise model for the syntax and semantics of the historically grown notation systems, taking the conventional way of notating musical dynamics as a simple example. It turns out that no single such model is possible, but a multitude of incompatibles: some have fundamentally different evaluation algorithms, others only slightly different parameter settings. Musical practice is allowed to switch between these models without even noticing their existence, but science may need distinctness. This article constructs and demonstrates an extensible mathematical framework for their precise description and proposes an extensible nomenclature system as a basis for their application and discussion.

## 1 Introduction

### 1.1 General aims and methods of the re-modeling approach

Musical notation can be and has been discussed and analyzed under very different aspects and theories. This paper concentrates on one single aspect: musical notation seen as a “precise language” in the sense of a system of well-defined graphical signs and rules for their combination, together with unambiguous rules for their interpretation, which allow to translate these signs and their spatial configuration to notions from some mental model and their relations.

In different concrete historical, practical or artistic settings, this aspect will have very different degrees of relevance. One extreme position may regard the graphic symbols contained in some piece of musical notation as mere inspirations and suggestions for free human improvisation. The opposite extreme aspires to totally automated execution of that graphic input. Most productive situations will take a position somewhere between these poles.

But whenever any kind of communication, memorization or textuality is assigned to musical notation, the aspect of a precise language can be helpful, regardless of how “precise” the real interpretation and execution finally will be: The analysis of that language as a mere symbolic transformation, as a transformation between two abstract, purely algebraic systems, will possibly show uncircumventable properties, which can be *neglected* by an interpretation, but never *negated*.

Whenever any cultural code is conceived as such a precise language, its syntax and its semantics must be describable precisely, i.e. by *mathematical* means. The technique of *mathematical remodeling* takes the informally given rules for notating some coherent aspect of music (here: dynamics) and tries to construct a pure abstract algebraic mathematical system, which shall come as close as possible to the interpretation rules applied in everyday practice.

The first and most important uncircumventable learned from this method is that there is no single system reflecting all practices, but a multitude of different variants with contradicting rules (see module “mn.intensitas.evalV” in Section 2.6 vs. module “mn.intensitas.simile” in Section 2.9). In practice, a pianist playing Chopin’s b-flat-minor sonata must switch between these fundamental different coding systems without even knowing their existence (see Section 2.9).

Substantially, the strict mathematical formalizations of the different models share a lot of common formulas and differ in details. This is clearly visible in the following text by the “import” and “replace” statements in the modules’ text, contained in the figures. Thus on the fly a classification grid is generated, as well as a naming convention for these different notation systems,–a truly novel result.

By applying a well-proven specification technique for *computer languages* (see Section 2.1 below), we transform the graphical input into a collection of domain-oriented mathematical data (see the corresponding figures in sections 2.6 and 2.10), or vice versa. But any concrete aesthetic “meaning” of this data is left totally open and must be defined by subsequent transformation processes. Our method is only intended as a *preparatory step* for clarifying the possible variants of notation and for eliminating methodological inconsistencies. The resulting data are meant as mere proposals to the domain experts in psychology, historic musicology, artistic practice, sound processing, etc.: They can select from the various interpretation systems, according to their needs, results, taste and experience. But this selection is from now on explicit and documented.

### 1.2 Structure of this article

In the following text we separate the phases of model construction and application clearly: The mathematical formulas are contained in figures and appear there without almost any comment of the intended usage.

In the flow text we apply these structures to phenomena in music notation, including historic examples and computer-based modeling frameworks.

Section 2.3 describes informally one particular and slightly simplified interpretation of notated dynamics. This is reconstructed as a basic mathematical model in Section 2.4. Section 2.5 applies this model to concrete examples and thus provides precise definitions of fundamental properties; Sections 2.6 and 2.7 define a first “V-analysis” of non-explicit fork ends; Section 2.10 defines and applies “DDQ-analysis,” which makes a first step in transforming the symbolic notation into some numeric linear domain.

### 1.3 Related work

Most musicological studies that deal with the phenomenon of music dynamics can be roughly divided into (a) empirical and (b) historical-hermeneutic oriented research. In the empirical field the focus is often on the relation of perceived loudness and acoustical correlates [1] [2] [3] [4] [5] [6] [7] [8] [9]; music dynamics from a neurological [10], therapeutical [11], or psychological [12] [13] [14] [15] point of view or music dynamics as a feature in music information retrieval [16] [17].

In the area of historical-hermeneutic oriented research, textbooks/instructions [18] [19] [20] [21] as well as the analysis of phenomenon concerning specific compositions, composers or epochs [22] [23] [24] [25] can be found frequently.

In contrast to all these, this paper deals with notation (of one single parameter) only as a completely symbolic operational system, mapping abstract signs to abstract contents.

Morris states that “temporal sequences are not simply one-to-one relations between sound and symbol, but complex, context-sensitive rule systems, which when mastered become second nature. If score reading is rule based, then notation serves in the function of language.” Thus, reading includes skills “completely transparent to the user.”[26] This article wants to make these (historically grown) transparencies a little bit more visible.

With one of the more recent papers there is some overlap: Grachten et al. 2017 [17] applied empirical-psychological modeling, based on machine learning, to sound perception of piano recordings. They verified empirically two different computing methods by comparing their results to a temporal curve, which was derived from the original notation. This curve resembles our approach, as it is calculated as a superposition of five or more different functions, each derived from one component in the notated score, including metric position, articulation symbols and dynamics.

Using the output curves from our DDQ transformation (see the corresponding Figure in section 2.10) as an input for *φ*_1_, *φ*_2_ and *φ*_4_ in their Fig 1 is a typical example how our analysis can serve as a preparatory step for research which combines notation and empirical data.

**Fig 1:**
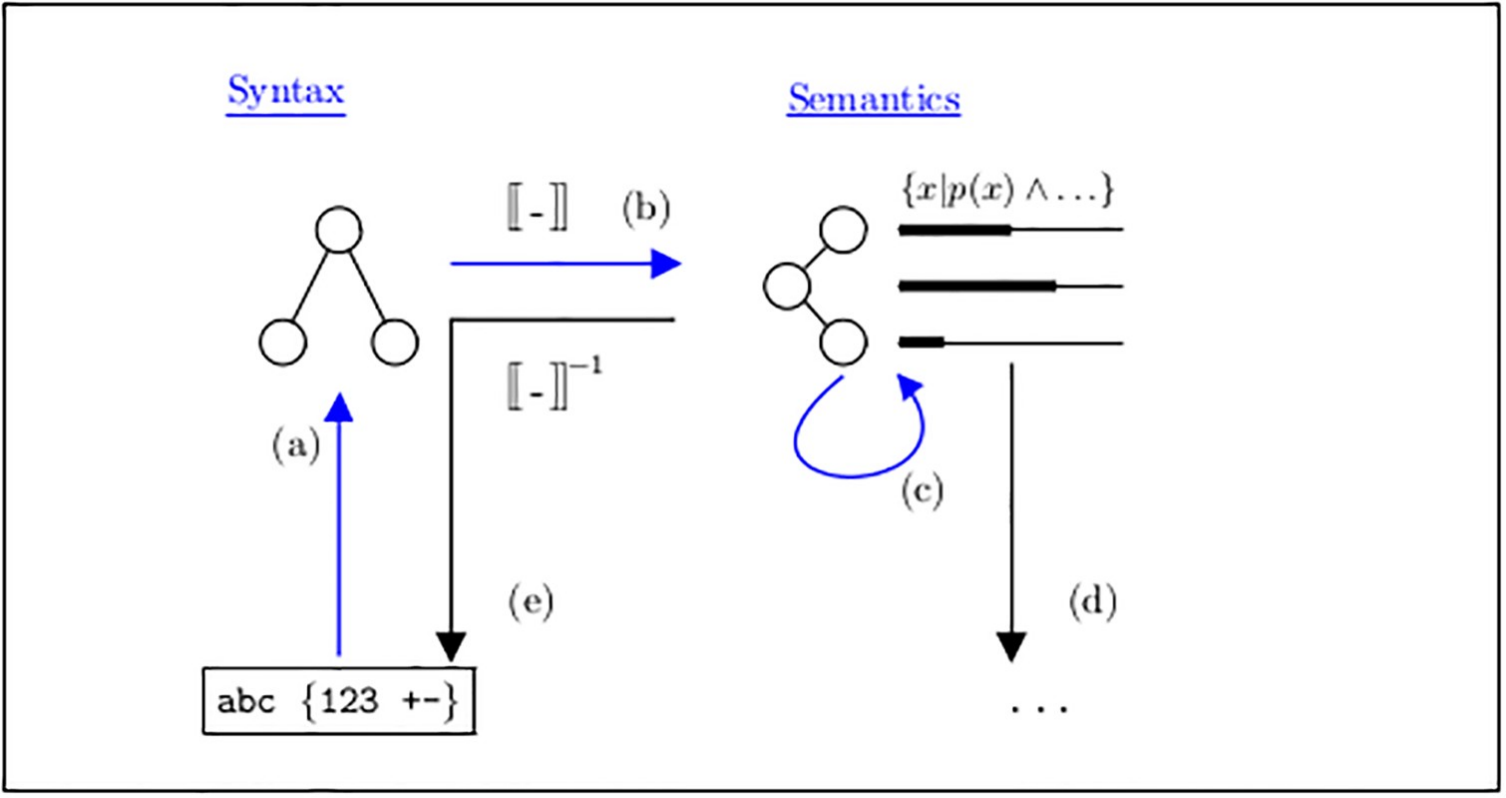
Translation processes in computer language architectures.

## 2 Re-modeling music dynamics

### 2.1 Mathematical models for computer languages

For the mathematical re-modeling of notation systems we adopt fundamental concepts from the fields of *computer languages*. These are languages designed specifically for human–machine interaction. They serve as so-called *external representations* of some mathematically defined data, used for input and output. In contrast to natural languages, their syntax is intentionally utmost simple, because they are designed only for this specific purpose.

The basic data flow and processing are shown in Fig 1: In a first step (a), some external representation, for instance, a text in some character encoding format and contained in some disk file, is “parsed” into a computer internal data structure which corresponds to the syntactic structure of the input text. Corresponding to the simple structure of the syntax, this is a simple graph in form of a directed tree.

In a second step (b) a so-called *semantic interpretation function* is applied, often written as double brackets 〚-〛. This translates the data into the *semantic sphere*, which is also populated by mathematical objects only, but with a much richer variety: Here all kinds of continuous and discrete mathematics can be applied, according to the needs of the subject of modeling like continuous functions, scalar data, also with physical units, automata, rewriting rules, and so on up to high-order constructs like constraint, inference and proof systems.

Once the input has been translated in the corresponding model in the semantic sphere, in step (c) further transformations and evaluations may modify the state of the model, by (d) the model can be used for controlling physical effects or generating further output in different formats, or (e) it can be re-exported by inverting the translation and parsing processes (b) and (a).

This meta-model has the nice properties that its theoretic fundamentals have been thoroughly analyzed during the last fifty years, and that it has been successfully applied in practice frequently. [27] [28]

The transfer to CWN implies that the syntactic sphere is represented by the graphic symbols of music notation and their relative positions, while the semantic sphere is a mathematical structure which represents the intended music structure.

We call the triple of the syntax definition, the structures living in the semantic sphere and the translation function, a *semantic triple*, because the three components together define the intended meaning of some aspect of graphical music representation.

The main advantage of this approach is the clear separation of both spheres: In most Musicology texts this does not happen and is not required. A phenomenon in notation can often be taken as a placeholder when speaking about the denotated musical content as a kind of metaphor, without obfuscating the meaning of the text. This is no longer the case when talking about notation itself: Notation is the entirety of rules translating front-end phenomena to intentions and vice versa. Before discussing the translation between these two spheres, we must clearly distinguish them.

Our experience with this approach has shown that the discussion of processes (a) to (c) is sufficient for defining precisely the meaning of notation, constructing all usual variants, finding uncircumventables and constructing a classification network.

The processes in (c) further include “computer aided composition” and “computer aided analysis”, while (d) includes “automated sheet music production” or “sound synthesis”. Once a model for the semantics of a particular slice of the music notation have been constructed by formalizing and analyzing according to path (a) and (b), there will be a more precise basis also for reasoning about paths (c) to (e). But this is out of scope of our current work.

### 2.2 Mathematical foundation

The formalization is based on the well-proven *Z language* [29]. Its principles are important not only for mathematical writing but for thinking about music and notation in general:

A *binding* is an object which enumerates a finite set of *identifiers* and assigns one particular *value* to each of them.

A *schema* is an object which enumerates a finite set of *identifiers* and states a finite set of *conditions* about the possible values of these identifiers. (Such a condition may restrict the value of one single identifier, or the allowed combinations of the values of several identifiers). Each schema is identified by a *name*.

The *extension* of a schema comprises all bindings which bind (at least) the identifiers of the schema and fulfill all of its conditions. The extension of a schema is always an *infinite* set of bindings, because infinitely many possible identifiers are (of course) not restricted by a schema.

Whenever a schema *A* is *imported* in another schema *B*, this claims that the conditions from schema *A* are fulfilled by all bindings in the extension of *B*. The identifiers introduced in *A* are visible in *B* and their possible values and value combinations can be further restricted, with or without referring to the identifiers newly introduced in *B*.

The step of importing a schema and adding further conditions on the bindings is called *refinement*; in practice it is executed repeatedly, building a *refinement chain*.

In our context, a particular schema (with the enumerated identifiers and all conditions on its values) can be seen as a model of one particular composition. The schema (a finite specification text) and the (infinite) set of all possible bindings are indeed both the very same object, seen from two different perspectives.

But this is only one interpretation in a whole continuum of more or less specific refinements. In fact, there is no qualitative difference between models for styles, epochs, genera, or individual compositions. A rather early schema in a (hypothetical) refinement chain can, for example, model “CWN notation as such”. Its extension, the set of all possible bindings, corresponds to all possible pieces of notatable music. A next schema in the refinement chain can model “CWN in the nineteenth century”; a more specific schema can model “Beethoven’s Fifth Symphony”; and an even more narrow schema can model “last evening’s performance of Beethoven’s Fifth Symphony”. The sets of possible bindings get smaller and smaller, but are still infinite.

The difference is only a matter of quantity because as the conditions increase, the possible bindings become less. But always the former are finite and the latter stay infinite in number.

It is clear that these modeling principles go far beyond mere mathematical notation, but they already imply a kind of *fundamental ontologic strategy*. In fact, this strategy corresponds closely to the ontology of a work of music as defined by Roman Ingarden: “[Das Musikalische Kunstwerk ist das] durch die Partitur bestimmte schematische Gebilde [zusammen mit der vollständigen] Mannigfaltigkeit von möglichen, ästhetisch zugelassenen Konkretisationen” [30]. (“[The work of music composition is] the schema defined by the musical score, [together with] the multitude of all aesthetically allowed concretizations of this schema.” The word “concretization” stands for one particular performance of that composition).

This statement is obviously a petitio principii; nevertheless, it has marked an important historic step in the theory of arts. The basic structure of this idea resembles that of *model theory* in logic, developed (among others) by the Lwów–Warsaw school of logic, to which Ingarden had a close personal relationship. The same basic idea is the standpoint of Kurkela [31]. Our work can be seen as an attempt at its continuation and concretization.

### 2.3 Modeled notation situation

In the following we construct a mathematical model which is inspired by a very basic version of notating *musical dynamics*. Dynamics in conventional notation express an intended change of intensity, loudness or energy when producing a sound, while all other “parameters” like pitch, duration or timbre should stay unaltered. The rules for reading this aspect of notation are taught to every child learning the recorder, are supposed to be well-known to anyone who can read music, and apply to almost all sheet music. The rules we want to formalize can be comprehended informally as follows:

1. There is only one single monodic voice.
2. This voice holds a sequence of events, and two neighboring events are easily distinguishable.
3. Only sounding events are allowed, not rests (/pauses).
4. Each explicitly notated intensity value consists of selected character combinations in a dedicated font, like ***pp*** or ***mf***; at the most, one of these is assigned to each event.
5. Additionally there are forks or “hairpins”, which connect the starting points of two different events and stand for a crescendo or diminuendo over all spanned events, including the first but not the last.
6. All explicit values are “sticky”, meaning they are valid for all following events, up to the next event with such a value, or to the beginning of a crescendo or diminuendo fork.
7. The same holds for all implicit values reached at the end of a fork.

Rule 3 is only a restriction of the scope of this article. All other rules are similar to those taught nowadays in elementary music education, but they are hardly ever explicitly published.

Trying to formalize these very simple rules yields surprisingly complicated results. Please remember that this set-up is only “an inspiration” for the modeling; the real application in musicology happens, as described above, a posteriori, by comparing its results with practical experiences. Moreover, significantly different (historic or contemporary) rule sets exist, which are treated in Section 2.9 below and which can be chosen as an alternative.

### 2.4 Basic model

#### 2.4.1 Voice and events

**Fig 2:**
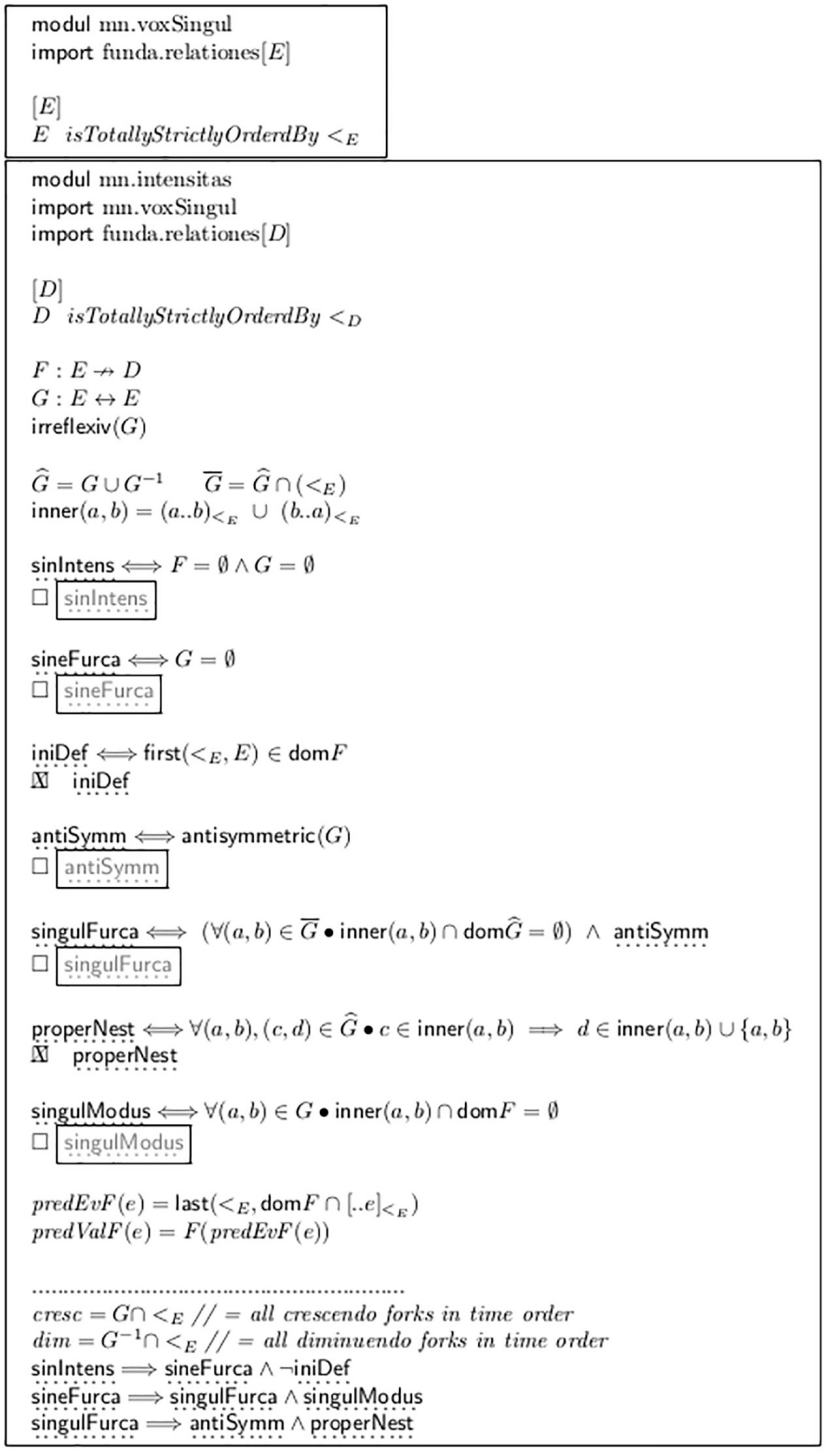
Mathematical re-modeling of events, dynamics and forks.

**Fig 3:**
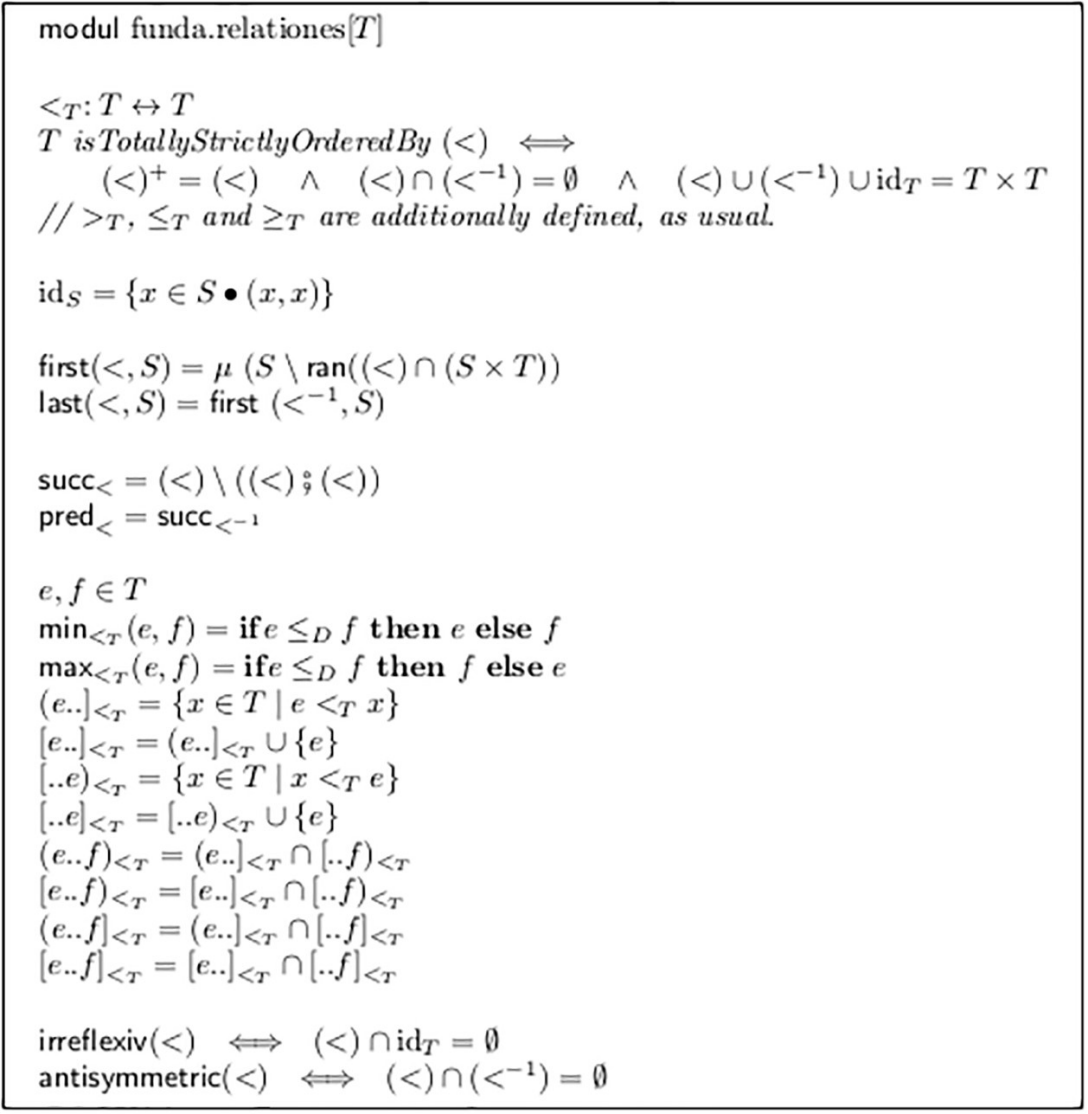
Basic notions about relations.

The basic syntax of our model is constructed completely by the formulas in Fig 2. It first defines a *module* named mn.voxSingul. This module *imports* a library module named funda.relationes, which defines basic facts about relations (see Fig 3). Due to this import, all definitions contained in this figure can be employed directly in the formulas.

Informally, the absolute mathematical behavior and the intended meanings of the objects defined in Fig 2 are as follows:

The set *E* is a *(finite) given set*. In the tradition of the *Z notation*, [29] a given set is a collection of discrete and distinguishable entities. We only know that these discrete and distinguishable entities do exist, that they are “elements of” that set, and that any two of such given sets are disjoint. All further properties about the sets and their elements must be added explicitly.

It is our intention to use the elements of *E* as the events of one notated monodic voice. But this intention in no ways plays a direct role in the mathematics, unless we model it by explicit properties.

Therefore we add the object <_*E*_. Its type is that of a relation on the Elements of *E*. This fact is written as <_*E*_: *E* ↔ *E*.

A relation between two sets *A* and *B* can be defined as a set of selected pairs, the first component of each pair from *A* and the second from *B*. (Here both sets happen to be same, namely *E*). Thus, each relation *r*: *A* ↔ *B* is a subset of the set of all possible pairs. This is written as *r* ⊆ *A* × *B*.

The simple notion of a relation, and the resulting algebraic structures are very powerful tools in the field of discrete mathematics. There are many sensible operations on relations, which create one relation from others, and predicates, which characterize a subset of all possible relations.

For example, the relation <_*E*_ is required to be a *total strict order* on *E*. This means that for any two elements *a*, *b* from *E* either *a* = *b* or *a* <_*E*_ *b* or *b* <_*E*_ *a* holds. And that the relation is *transitive*, so if *a* <_*E*_ *b* and *b* <_*E*_ *c* then also *a* <_*E*_ *c*. Please note that these properties are defined, as is typical for mathematical abstractions, totally independent from their application purpose and can be reused in very different contexts. Therefore they are defined in the imported module from Fig 3.

When applied to a finite set (which is the case here), further consequences are the uniqueness of a successor of a given element (i.e. a larger element, which is smaller than any other larger element), and a lowest (first) element in a finite set. This is defined in Fig 3 as succ and first, together with the counterparts pred and last.

In our model, <_*E*_ stands for the temporal order of the music events from *E*.

#### 2.4.2 Explicit dynamics and forks

Similarly, the second module mn.intensitas in Fig 2 defines a given set *D* and a total and strict order <_*D*_. The set *D* shall model the different steps of a dynamic scale, like

$$ppp{<}_{D}\;pp{<}_{D}\;p{<}_{D}\;mf{<}_{D}\;f{<}_{D}\;ff{<}_{D}\;fff$$

(This usual but informal way of abbreviated writing shows the *successor* relation, which unambiguously induces the total order <_*D*_ if and only if all elements of the set are mentioned).

This module imports mn.voxSingul, so that *E* and <_*E*_ can be used.

The set *F* is a *partial map* from events to dynamics, so it can model the intensity values assigned explicitly in our monodic score to selected events.

The set *G* is intended to model the set of all appearing *forks*, (also called *hairpins*), which are graphic line symbols for *crescendo* and *diminuendo*. Each pair (*e*_1_, *e*_2_) ∈ *G* states the existence of a fork entity whose sharp end is aligned to (the starting time point of) *e*_1_ and whose open end is aligned to (the start time point of) *e*_2_. (The set *G* may also represent textual renderings like “*cresc* − − − −”, as long as they have unambiguous horizontal extent, aligned with two events, comparable to that of a fork).

As a consequence, a fork cannot start and end at the same event, because it would have neither extension nor direction. This is modeled by the explicit irreflexiv condition in the definition of *G*. (As a further consequence, no fork can start on the very last event in the sequence *E*. If a particular given score ends with a diminuendo spanning the very last note, then the model needs one final “dummy” event to be appended. Ending with a diminuendo is quite frequent (see “morendo” at the end of Mahlers Symphony No. 4), and Nielsen’s No. 4 ends with an “unterminated” crescendo.

Between any two events there can be maximally two forks, one crescendo and one diminuendo. This is *implicitly* given by the fact that we use a *relation*, which is a *set* of pairs: Each particular thing may be contained (or not) in a particular set, but it cannot be contained more than once. If we wanted to support such a “multiple fork” notation, we would have to choose other mathematical building blocks, like multisets.

**Fig 4:**
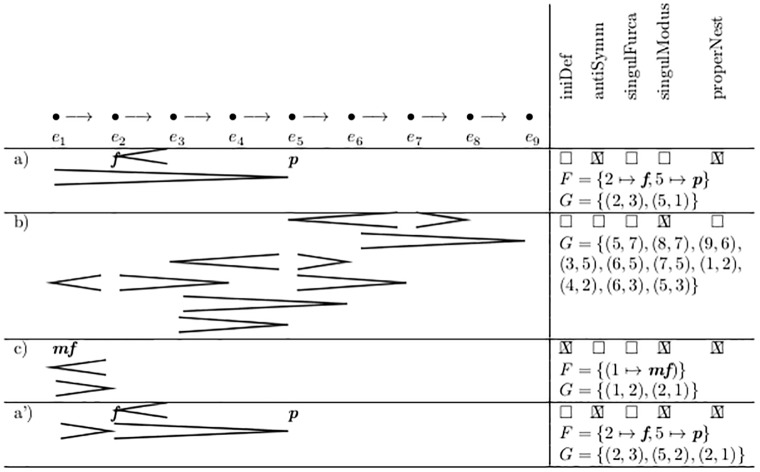
Syntactic structures of intensities and their properties.

Fig 4 shows some examples of volume assignments and the corresponding values of *F* and *G*.

#### 2.4.3 Preparatory and simplifying transformations

For any practical application to a given notated piece of music, an instance of this data model must be constructed as an abstraction, by some preparatory steps: First, polyphone settings must be split into several monodic sequences. Each appearing dynamic value must be assigned unambiguously to (the start point of) exactly one event. The same is true for both ends of each fork. The scale of dynamic values *D* and the relation <_*D*_ must be defined. The names of the values in the example above are totally arbitrary and have been chosen only for illustrative purposes; mathematically they could be called *d*_1_ <_*D*_ *d*_2_ <_*D*_ *d*_3_ etc. In practice, more than one concrete front-end wording can stand for the same value from *D*, for example, when defining ***mp*** = ***mf*** or “*sotto voce*” = ***p*** or “più ***p***” = ***pp*** or “*flüchtig*” = ***ppp***. The same holds for “*morendo*”, “*smorzando*” etc., standing for “*diminuendo al niente*”.

With each concrete example, each of these preparation steps may turn out to be trivial or critical. But they must be executed to make our model applicable. However these definitions are met, the following analytical results are uncircumventable.

#### 2.4.4 First derivations and properties

For first calculations some auxiliary data are derived: $\hat{G}$ contains all event pairs which are connected by a fork, in any direction; $\bar{G}$ contains only those pairs which are in temporal order. Thus, any pair (*e*_1_, *e*_2_) ∈ *G* which is also $\in \bar{G}$ is a crescendo from *e*_1_ to *e*_2_. If it is not $\in \bar{G}$, then instead $\left(e_{2},e_{1}\right)\in \bar{G}$, and it means a diminuendo from *e*_2_ to *e*_1_.

For example a) of Fig 4 we get $\hat{G}=\left\{\left(2,3\right),\left(3,2\right),\left(5,1\right),\left(1,5\right)\right\}$ and $\bar{G}=\left\{\left(2,3\right),\left(1,5\right)\right\}$. This is additionally realized by the definitions of the auxiliary sets *cresc* and *dim* at the bottom of Fig 2, which hold all crescendo and diminuendo forks in temporal order.

Now first and trivial *classification properties* can be defined quite naturally. For each of them the very first two appearances of their names in Fig 2 have the following roles: First the meaning of the property is defined by a logical equation in the form *p* ⇔ *e*: If the property *p* is true then the complex expression *e* is also true, and vice versa.

In the next line, the property appears again, standing alone and preceded by a *checkbox* ▫. This means that the property can be “switched on or off”. The frame around it puts it *outside* of the module’s text (see the box around the module text as a “box with holes”). When the check box is activated, the frame disappears, and the statement becomes part of this particular model instance. This is shown as an example in Fig 2 for the standard combination iniDef and properNest. Whatever the concrete values bound to the identifiers are, from now on only combinations which fulfill this property are included in the extension of the constructed schema.

In a later digital publication, these checkboxes shall be implemented as really interactive, automatically checking the implications like those described below. Here, in the paper version, it shall indicate to the reader that this property is a dimension of the classification network: It may or may not apply to this particular model instance.

sinIntens reflects the fact that the modeled score contains neither dynamic values nor forks; sineFurca means that no forks are contained.

iniDef means that the very first event of the voice is assigned an explicit dynamic value. The predicate first() is again taken from the imported library module: in Fig 3 the fact that an element is the first in a given set *S* w.r.t. the order <_*T*_, is declared equivalent to the fact that no “smaller-than-arrow” of this relation starts in *S* and ends at this element.

antiSymm means that both a crescendo and a diminuendo fork never connect the very same events. This property of music notation is modeled in a very simple way, because the corresponding property antisymmetric is standard in the theory of mathematical relations. (We use English words for standard notions from mathematics, and Latin for those from musicology).

As long as “notation” means “traditional sheet music”, this property is always fulfilled; in the examples in Fig 4b) and 4c) it is not.

The events *spanned* by a fork (*a*, *b*) are given by the set inner. This is defined in Fig 2, based on the general definitions of the different types of intervals (open, closed, half-open) from Fig 3. Property singulFurca signifies that no fork starts or ends at an inner event. The property singulModus signifies that no inner event carries any explicit dynamic value. (= any event gets an intensity at most by being spanned by [one or more] forks, or by an explicit value from *D*).

The property properNest signifies that if a fork does start at an inner event, then the other end of the fork is also an inner event, or aligned with the spanning fork. This property is crucial for many evaluation algorithms and fulfilled by most sheet music. In Fig 4 only line b) does not.

The lines in Fig 2 below the dotted separation line show some implications between properties. They do not add anything to the model substance either, as they are simply tautologies: Their being true can be derived from the combination of the definitions. Nevertheless their explicit enumeration may be helpful for human readers and for further discussion.

#### 2.4.5 Counting fork nesting levels

**Fig 5:**
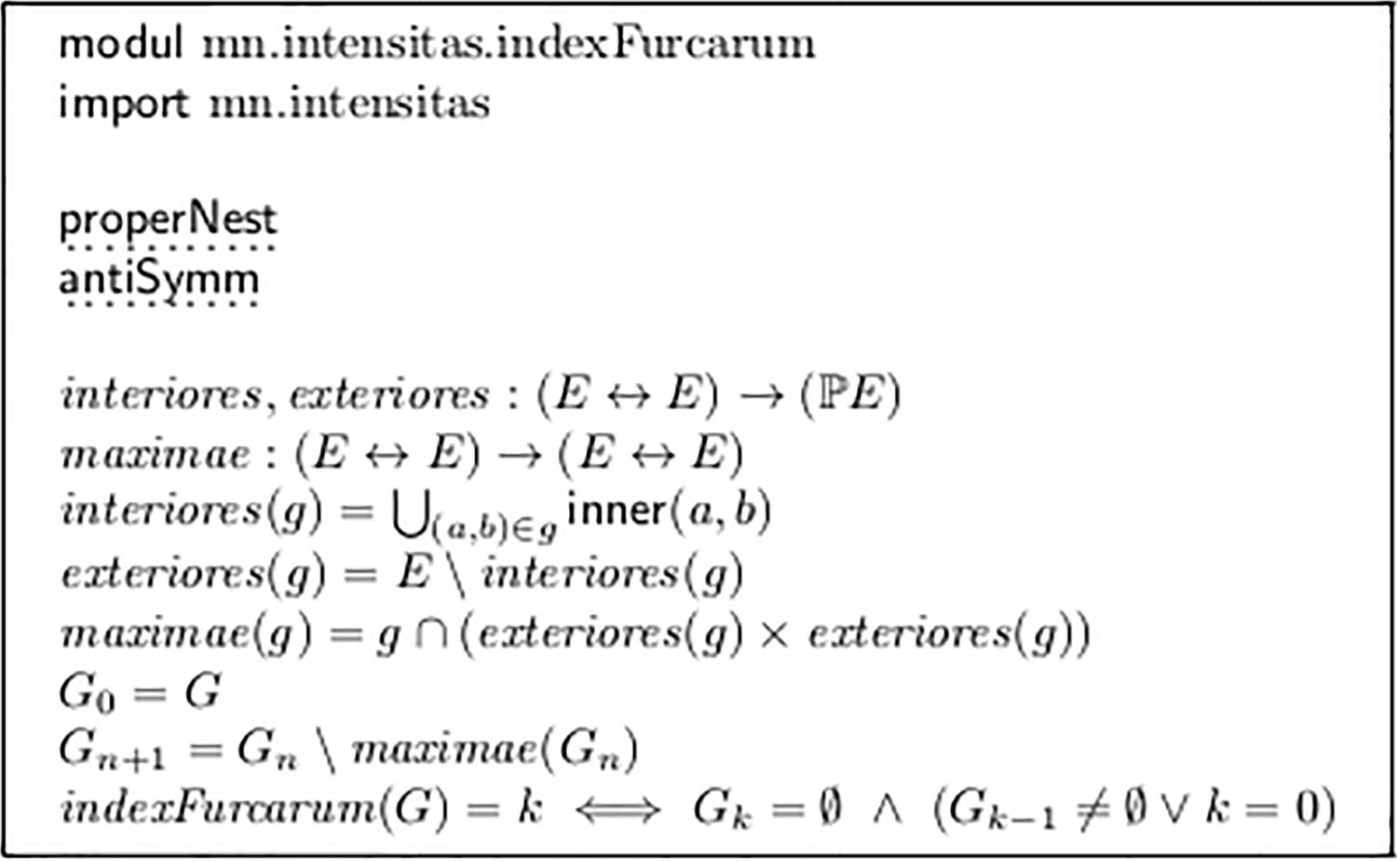
Counting fork nesting levels.

If properNest and antiSymm are fulfilled, singulFurca can easily be refined to *counting the nesting level* of forks, as realized by function *indexFurcarum* in module modul mn.intensitas.indexFurcarum in Fig 5.

*Maximae* delivers the set of all those forks the ends of which are not spanned by another fork, that is, those which are top-most in the forks’ stacking order. So the sequence *G*_0_, *G*_1_, … removes these forks and eventually becomes empty, which immediately gives the desired index.

This property is important, as values >1 are often required in practice: See for example the last bars of the Chopin example below, which establish *indexFurcarum* = 2. (Taking into account the envelope curve of the instrument’s physical sound production, we have even *three* stacked volume curves).

All following transformations are restricted to singulFurca, which means *indexFurcarum* ≤ 1. The treatment of nested forks seems a major effort, probably solvable only at a later stage of transformation. A possible strategy is to apply the following transformations to *maximae*(*G*_0_), temporarily ignoring the sub-ordinated forks, and to re-enter them into the evaluation chain at some later point.

### 2.5 First applications to music

All movements in “Das Wohltemperierte Clavier” have property sinIntens. The oboe voice of Aria #39 “Flößt mein Heiland” from BWV 248, as a Baroque echo aria, is a typical example of ¬sinIntens ∧ ¬iniDef. The first dynamic mark “piano” does not stand with the very first note but with the first “echo” in the oboe solo. Section 2.6.1 will show how to deal with this situation in a later step of the interpretation chain. Of course, all J.S. Bach’s works fulfill sineFurca.

Because of its omnipresence, the property antiSymm is likely to be overlooked. But it is nevertheless important to make it explicit because, indeed, there are cases in which it does *not* apply:

Case (A) occurs when displaying analysis results. Consider a “dynamic sum”, similar to a “rhythmic sum”: Fig 4b) shows all forks from op.16 by Schönberg, second movement, in the five measures after rehearsal mark (4), simplified in rhythm and projected to one single voice, for analysis purposes.

(The vertical stacking order in our graphic corresponds roughly to that of the original score, as does the notation of the set *G* in the right column. Mathematically, this is of no significance, because the mathematical construct “set” always ignores the sequential order of any explicit enumeration of its elements, as well as possible repeating values therein).

The result obviously violates the properties antiSymm and properNest. But even more, the *systematic* violation of these properties can be seen as a hint that one major historic contribution of this work, the important step towards systematic treatment of “Klangfarbenmelodie”, is not only done in the famous following movement, but already plays a role here.

A very practical consequence is that most conventional modeling frameworks (like LilyPond, Finale, etc). *cannot* be used to capture and render the results of such a projection. Such a rendering could be desirable for teaching or publishing purposes.

Case (B) of violating antiSymm occurs in an *aleatoric* composition which gives some interpretation bandwidth to the player. Rules such as

- play any non-contradictory selection of forks, or
- play any non-contradictory and maximal selection of forks, or
- play any non-contradictory selection of forks and dynamic values, which contains at least 30 percent of the written text, etc.

give perfect sense to the situations in Fig 4a) to 4c). Again, such a composition cannot be written down with standard notation software either, due to the violation of antiSymm.

One could be tempted to argue that these texts are “only a pre-notation”, which are transformed into “valid CWN” only by the interpretative act of the player. But the semantics of any notation are always defined by a *chain of transformations*, as we will see in the sequel. Without an objective (structural) criterion, this labeling is just a petitio principii and, as such, illustrates well our central problem of defining notation.

Case (C) is a score for the realization of a piece of musique concrète, in the style of the Sixties. Fig 4 line c) can be read as: Take the recoding of a impulse formed instrument (Piano or Marimba), which has a natural diminuendo, and compensate it by a crescendo of the electric amplification.

But indeed, almost all of conventional CWN sheet music fulfills properNest and antiSymm.

### 2.6 A first step towards semantics: V-analysis of fork ends

Please note that up to this point the data model and also the derived properties can be seen as “mere syntax” in the sense of Section 2.1 above.

In a first step towards some “semantics” we model the *propagation* of the syntactically notated intensity values, and check for the *definedness* of those events which do not carry an explicit value in *F* but are the end of a fork in *G*.

**Fig 6:**
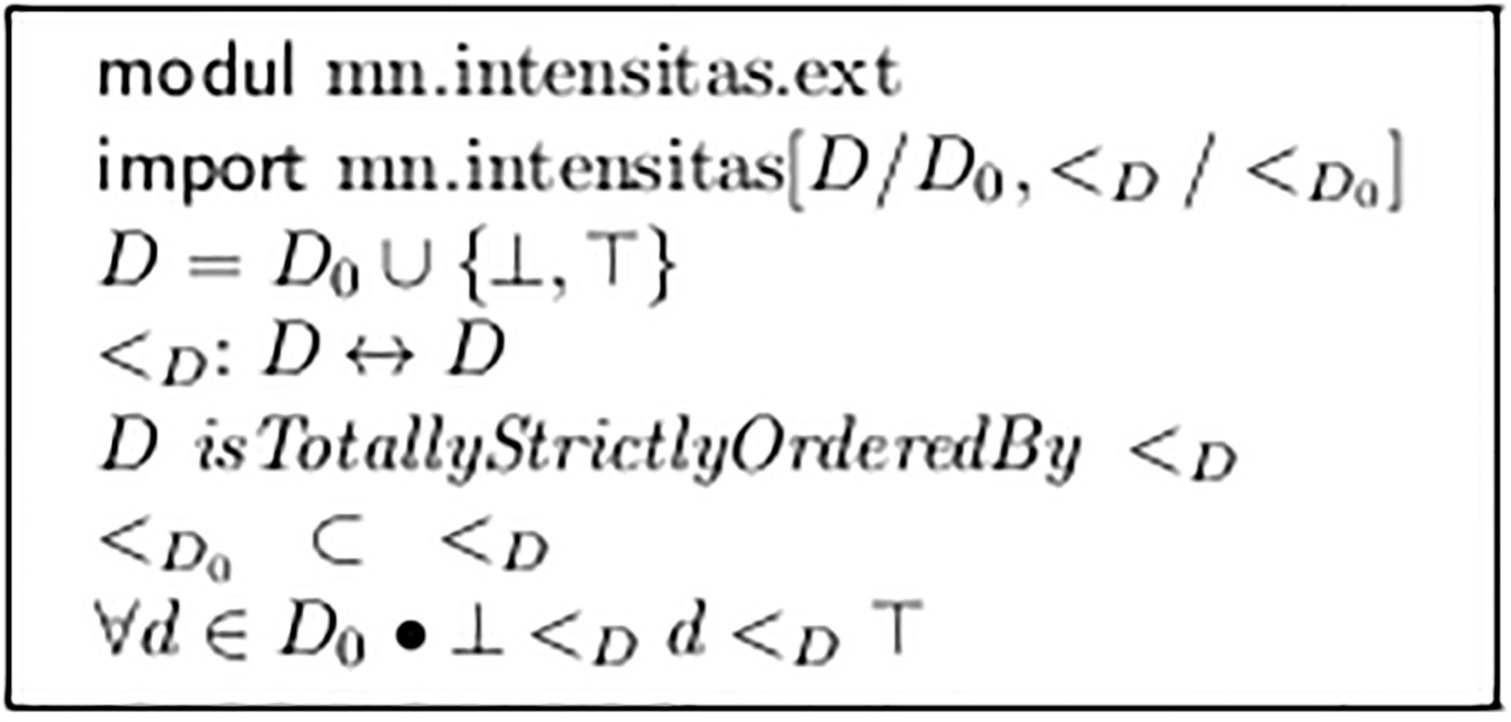
Extending *D* by top and bottom values.

**Fig 7:**
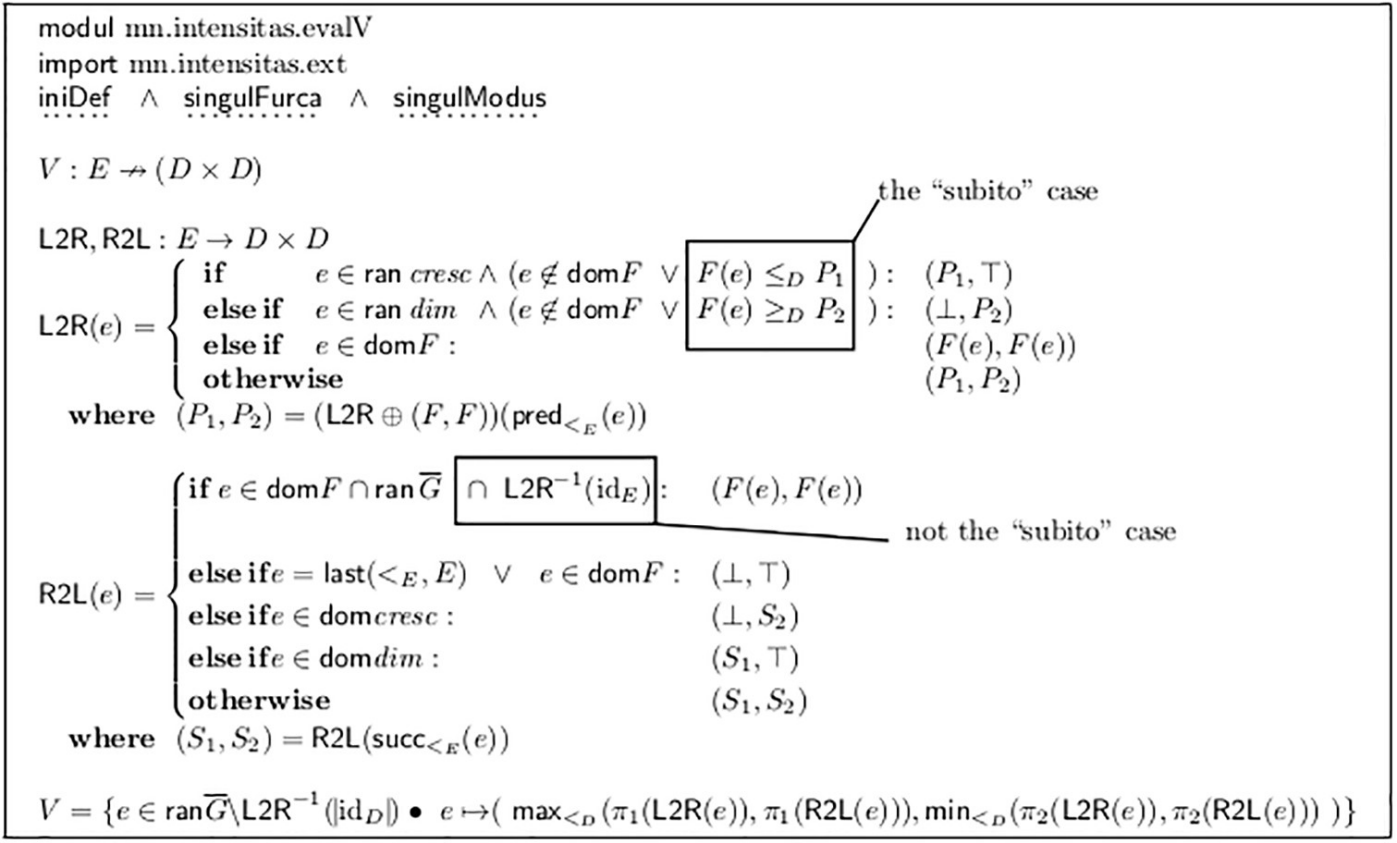
V-Analysis of fork ends, algorithm.

For this purpose, the set *D* and the order relation >_*D*_ must be enhanced by two additional synthetic values for “arbitrary low” = ⊥ and “arbitrary high” = ⊤. This is done by the module *mn.intensitas.ext* in Fig 6. Fig 7 shows the algorithm of the V-analysis.

Additionally the properties iniDef, singulFurca and singulModus must apply.

The result of this analysis is the map *V* which contains all those fork ends which do not have an unambiguous value, and maps to them the exclusive limits, that is, the values which definitely must be overtaken and underrun at the end of this fork.

Two arrays of 2-tuples L2R and R2L are filled, which collect the lower and upper (exclusive) bounds that are propagated from left to right and from right to left. This algorithm can be implemented efficiently: first filling L2R from left to right and then R2L from right to left is done in *O*(*n*), with *n* the number of events.

The propagation from left to right works as follows:

If a crescendo fork ends at event *e* and no explicit dynamic value is defined by *F*(*e*), then the lower limit of the preceding event is inherited but the upper limit is undefined = ⊤.

**Fig 8:**
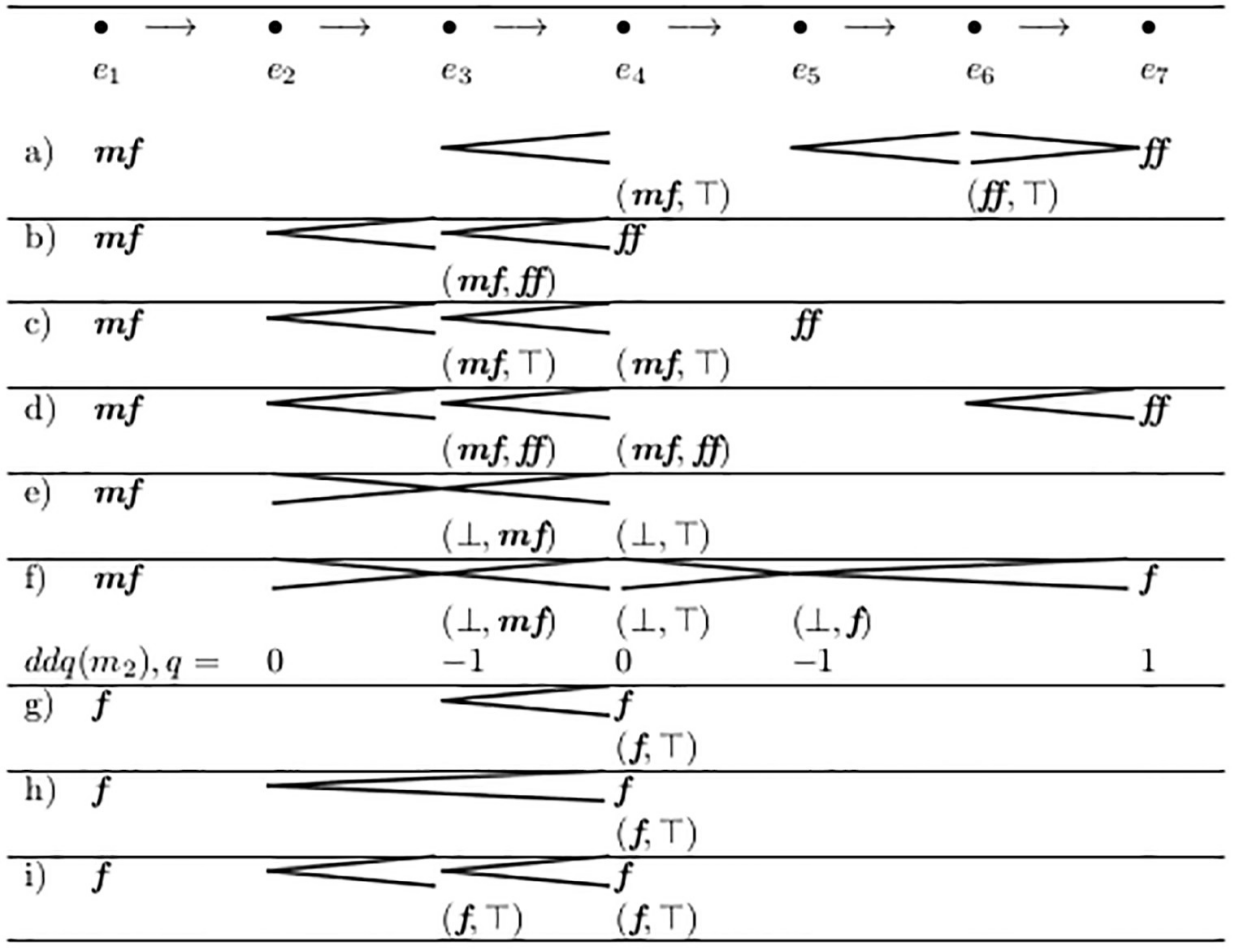
V-analysis of fork ends, examples.

**Fig 9:**
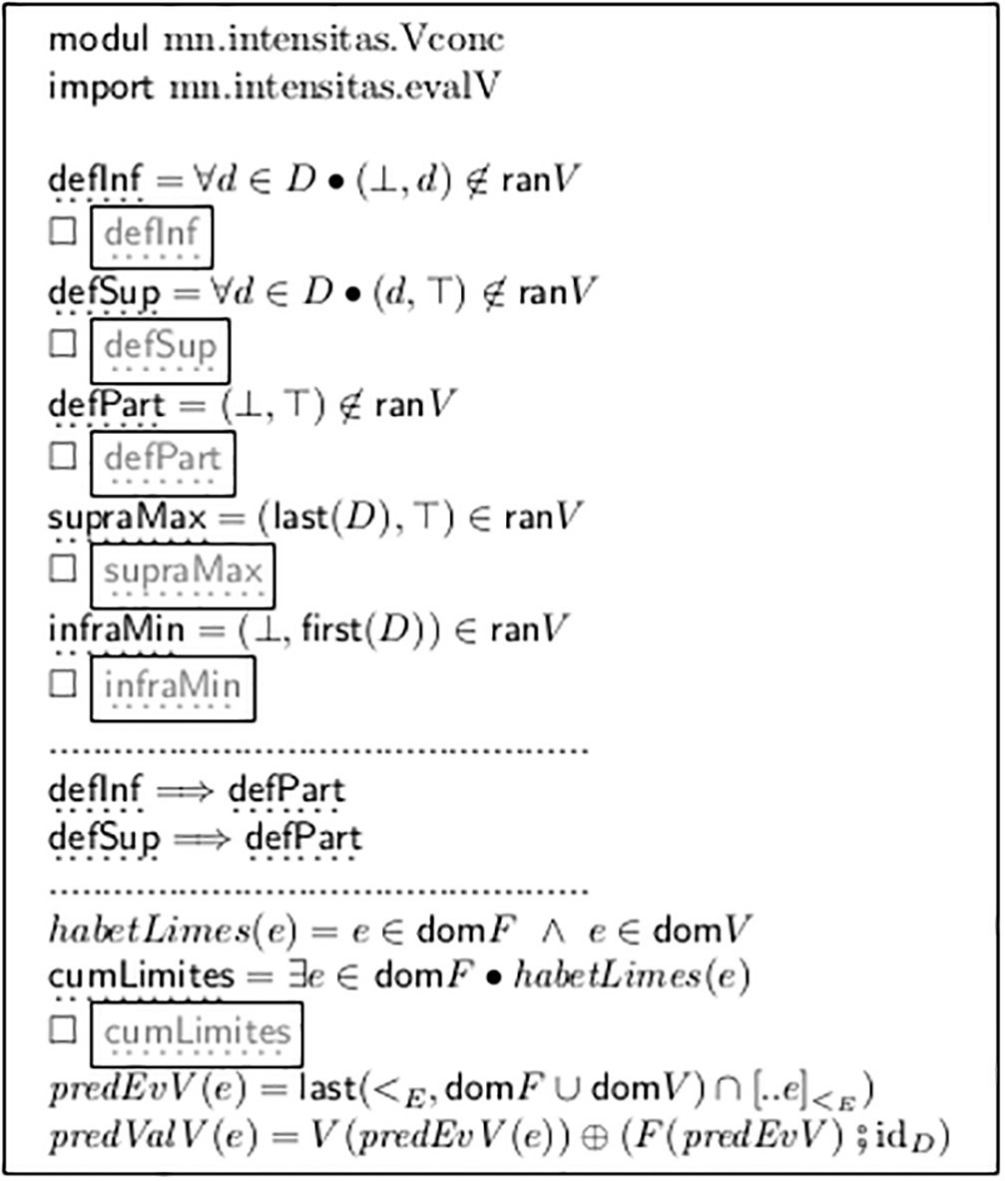
Conclusions from *V*.

An important exception is the *subito case*: If there is a value *d*_*e*_ in *F*(*e*) but the lower limit of the preceding event is equal to or higher than *d*_*e*_, then this value cannot be the target of the crescendo (see Fig 8, Lines g) to i)). In these cases we can think of a kind of *limes event* $e_{4}^{\prime }$, which stands for the time-point arbitrarily short before *e*_4_. At this time-point, the end value *d*_*x*_ > *d*_*e*_ = ***f*** of the crescendo must be reached, and the *d*_*e*_ = ***f*** assigned to the starting time of *e*_4_ is a “***f*** subito”. The pair at *V*(*e*_4_) is abused to hold the limes values for this time point $e_{4}^{\prime }$. This can easily be detected by the predicate *habetLimes*(*e*_4_), as defined in Fig 9. In all such cases, the pair in *V* is always of form (*d*, ⊤) at the end of a crescendo, or (⊥, *d*) at the end of a diminuendo. This important special case clearly shows the asymmetric nature of music notation.

The end of a diminuendo fork is treated accordingly.

Otherwise, if there is an entry in *F* this is copied onto both limits in L2R. This signifies a defined dynamic value which is not the subito case. Otherwise the values of the preceding event are inherited unchanged.

The propagation from right to left works as follows:

If an explicit value is assigned to *e* and a fork *ends* there, and that value is not the subito case, then this value is taken for both limits. Otherwise, if an explicit value is assigned (and no fork ends or it is the subito case) then both limits are undefined. These two rules model the fact that in Fig 8 ***ff*** is propagated to event *e*_4_ in Line d), but not in Line c). This again is an asymmetry in music notation.

Otherwise, if the event is the *start* of a crescendo(/diminuendo) fork, then the upper(/lower) limit of the following event is inherited and the lower(/upper) limit is undefined. Otherwise the values of the following event are copied.

Finally, the resulting map *V* is simply the conjunction of both pairs of limits. at all events which are the end of a fork and do not carry a (non-subito) dynamic value.

**Fig 10:**
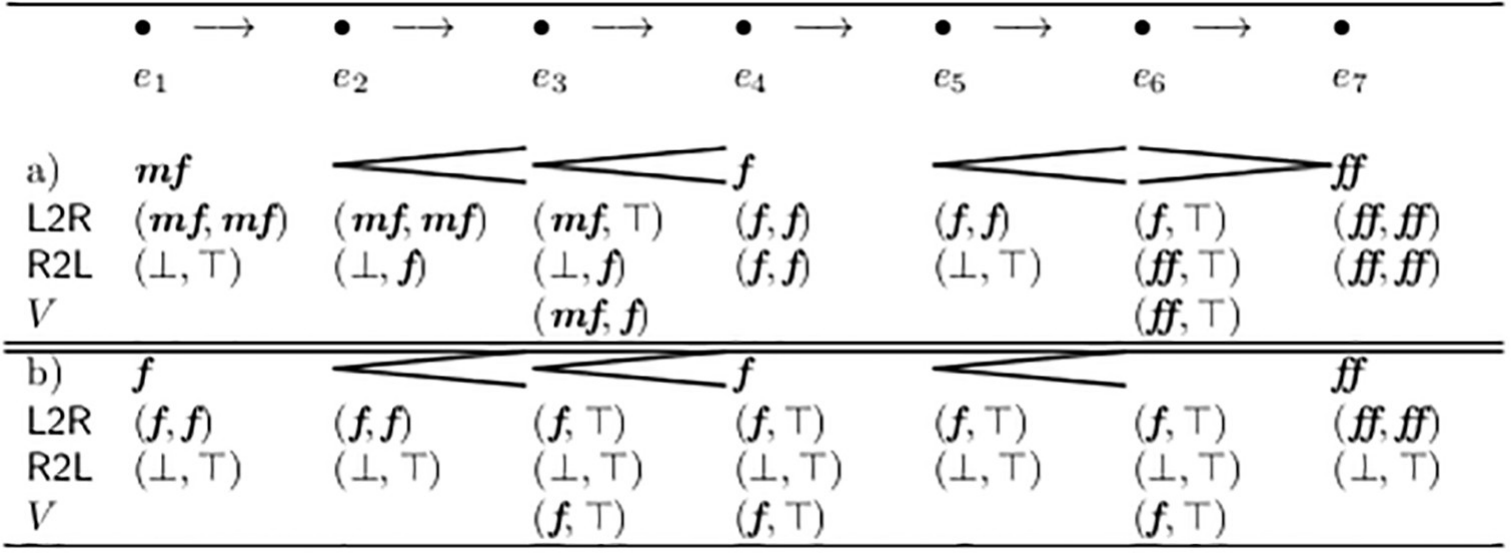
Examples of V-analysis calculation.

Fig 8 shows typical results of this analysis and Fig 10 demonstrates the operation of the algorithm.

#### 2.6.1 Transforming a model to make V-analysis applicable

The algorithm in module mn.intensitas.evalV (Fig 7) requires the initially listed properties. If a model lacks them, it can possibly be transformed accordingly to make V-analysis applicable.

**Fig 11:**
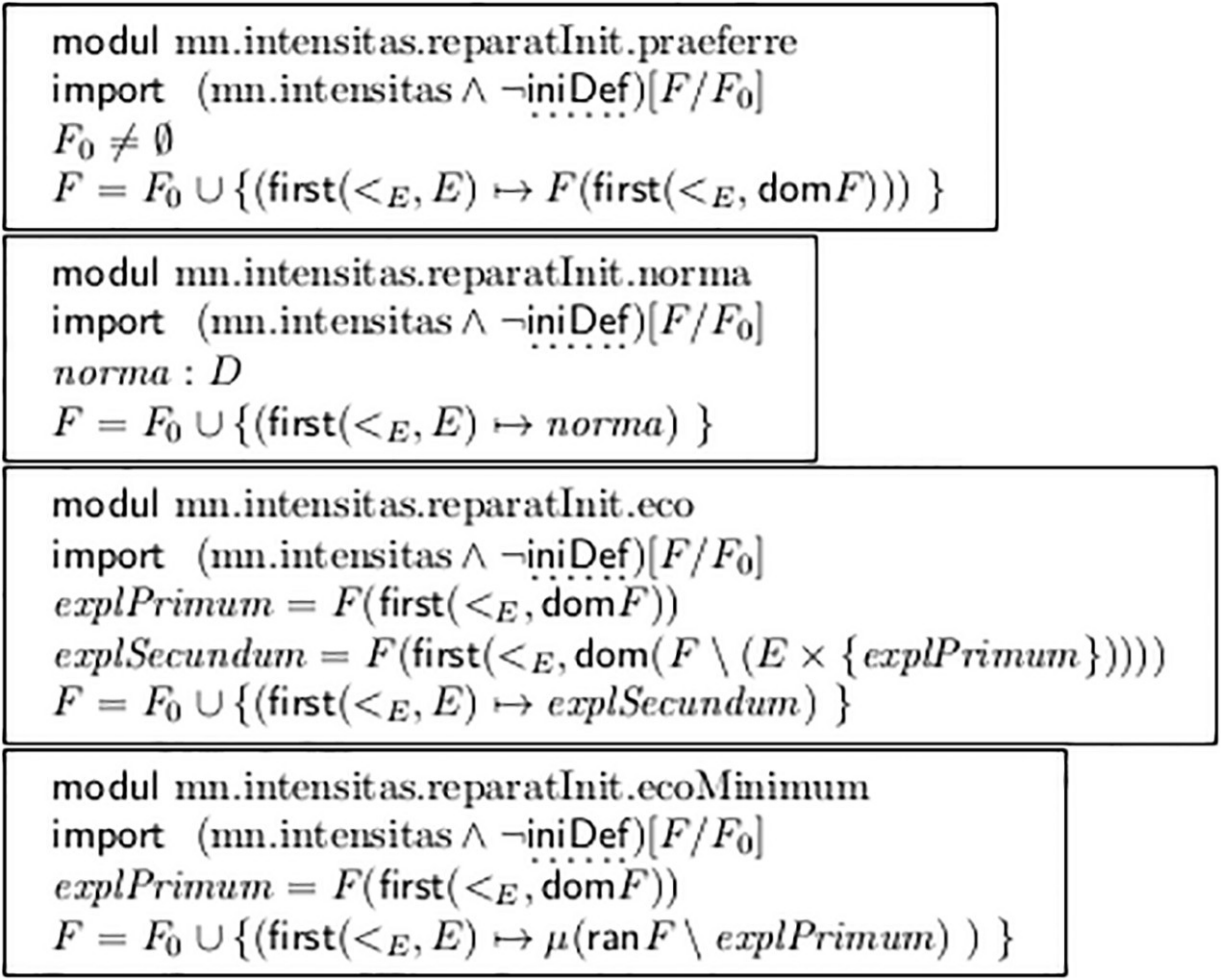
Repairing missing initials.

Fig 11 offers several solutions for repairing ¬iniDef = a missing initial dynamic value:

Mn.intensitas.reparatInit.praeferre simply copies the first explicit dynamic entry to the very first event. This raises a typical question: Why did the author not put it there? What is the meaning of its being delayed?

In certain epochs, styles and genres the method Mn.intensitas.reparatInit.norma seems to be employed frequently:

Mn.intensitas.reparatInit.norma ∧ [*norma* = ***f***] has been applied to the scores of the Mozart piano concertos, when extracting the instrumental voices. It also applies to many Baroque arias. Contrarily, an initial ***p*** is always explicit.

Mn.intensitas.reparatInit.norma ∧ [*norma* = ***p***] models the default for slow movements in certain styles, see the missing ***p*** at the beginning of the Adagio of Beethoven op.13. (All these details are out of scope of this article, which only wants to provide the vocabulary).

Mn.intensitas.reparatInit.norma ∧ [*norma* = 0x40] is applied by contemporary MIDI sequencer programs, taking the value 64 as the middle of the MIDI “attack values”.

More interesting is the method Mn.intensitas.reparatInit.eco, which can treat Baroque echo arias correctly, like the above-mentioned Aria #39 from BWV 248.

The first dynamic assignment happens somewhere after the beginning and starts the “echo” section; the second assignment switches back to “normal mode” and thus carries the value which is missing (in this style and epoch!) at the very first event. The code solves the problem, but it is not trivial, because it must ignore a redundant repetition of the first explicit value (as really happens in that aria in measure 78).

An alternative solution ecoMinimum only works if there are at most two elements in *D*, which is also not the case in that aria (***pp*** in measure 46). (The operator *μ* picks the one and only element from a set, and fails if the set’s cardinality ≠1).

**Fig 12:**
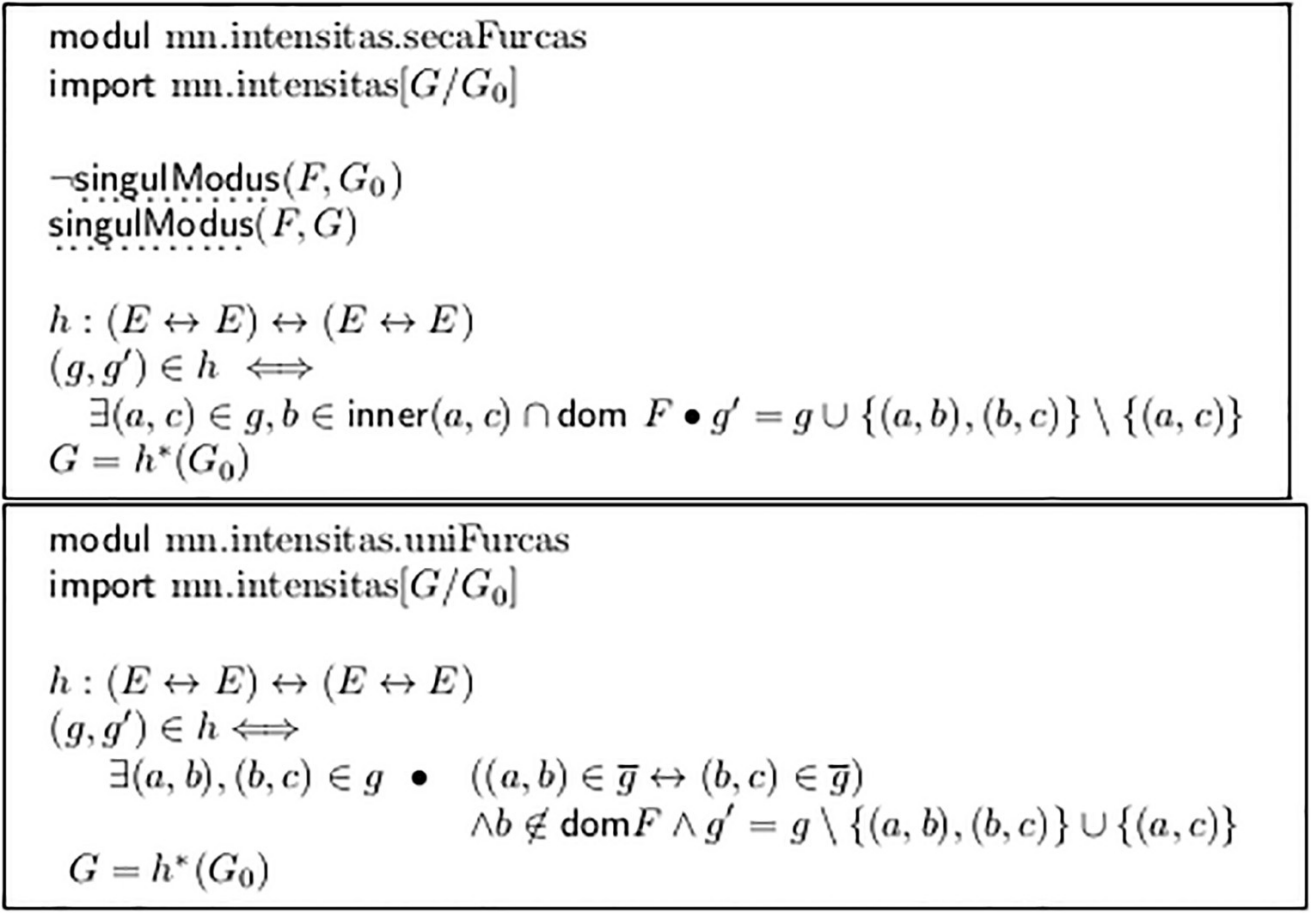
Transformations of forks.

Module mn.intensitas.secaFurcas in Fig 12 splits one fork into two at all points where an explicit dynamic value is spanned. Thus it establishes singulModus. (Fig 4 line a’) shows its application to line a)).

If we apply this function we gain application possibilities for other functions, but of course we *must memorize* that we have transformed the original syntax and that the original input to the transformation chain did not have this property, in case a later interpretative step (= semantic triple) assigns a different meaning to two adjacent forks than to one, as in *ddq*(*m*_1_) defined in Section 2.10.

### 2.7 Conclusions from the V-analysis of fork ends

The result of the construction in Fig 7 is the map *V*. It indicates (a) all fork ends which are under-specified and (b) all events which need a preceding *imes event* as the endpoint of the preceding fork. This corresponds to the main interpretation problems in the majority of classic/romantic sheet music. Module mn.intensitas.Vconc defines the corresponding properties of a model instance as a whole.

The contribution to the semantics related to one particular event is modeled in Fig 9: *predEvV* gives for every event *e* the latest predecessor of its successor which has an entry in *F* or *V*. If this is in *F* then *predValV* gives an exact and explicit dynamic value for *e*, encoded as a pair (*d*, *d*). Otherwise there is an entry (*i*, *a*) in *V* and *e* (if not spanned by a fork) must have some value between *i* and *a*, exclusively. If there is an entry in both, then this is a “subito” event.

Whenever we want to *execute* a particular piece of music, concrete initial intensity values must be given to each event. (Additionally, if the instrument is capable of continuous volume control, intensity curves should be defined for the duration of each event). For this, a chain of further transformations is necessary.

All entries (*i*, *a*) ∈ *V* with *i* ≠ ⊥ ∧ *a* ≠ ⊤ need only some *interpolation* between both limits (see Line b) and d) in Fig 8). The kind of interpolation does not necessarily differ from that which is required anyhow, namely for all events spanned by a fork.

The entries (⊥, *a*), (*i*, ⊤), and (⊥, ⊤) are fundamentally different. They require *extrapolation*. The absence or existence of those entries is reflected by new properties defined in Fig 9.

#### 2.7.1 SupraMax and infraMin

At the beginning of our model construction the set of dynamic values *D* has been simply stated as an arbitrary “given set”. In a concrete application we could collect all explicit dynamics appearing in one particular score and make them *D*. In any case, the properties supraMax and infraMin reflect whether there is a crescendo starting with the highest value in *D*, or a diminuendo with the lowest, respectively.

This can make an important difference in practice, for instance for sound checks, for sound synthesis, or for a conductor planning the execution dynamics for some longer orchestral movement. When supraMax applies, then the highest explict dynamic value appearing verbatim in a score is *not the highest notated*, because a crescendo starting there has a higher end point, and is also part of the notation. Thus, it makes also a difference in the sphere of theory.

Both properties can be removed by inserting a further highest respective lowest value into *D*, before it is enlarged to $\bar{D}$. But if not, one has to deal with the reality of a diminuendo with a non-writable end value,—as in the title of this article. It is a complicated and still open question whether the directive “diminuendo al niente” always means ⊥ as its end point, or a value niente ∈ *D* immediately above, which is special as it does not allow a diminuendo to start there.

### 2.8 Local repairs of partially undefined limits

A possible next step towards performance in the processing pipeline is to *repair* the unspecified fork ends.

Such a repair is possibly carried out by an experienced instrumentalist on the fly, perhaps without even noticing it. Then the reparation is part of musical practice, and highly determined by stylistic conventions, instrumental tradition or even personal taste.

Since all dynamic values must be defined for any execution, even mere technical systems must carry out these reparation steps, like music modeling frameworks with an integrated playing facility or midi file export.

**Fig 13:**
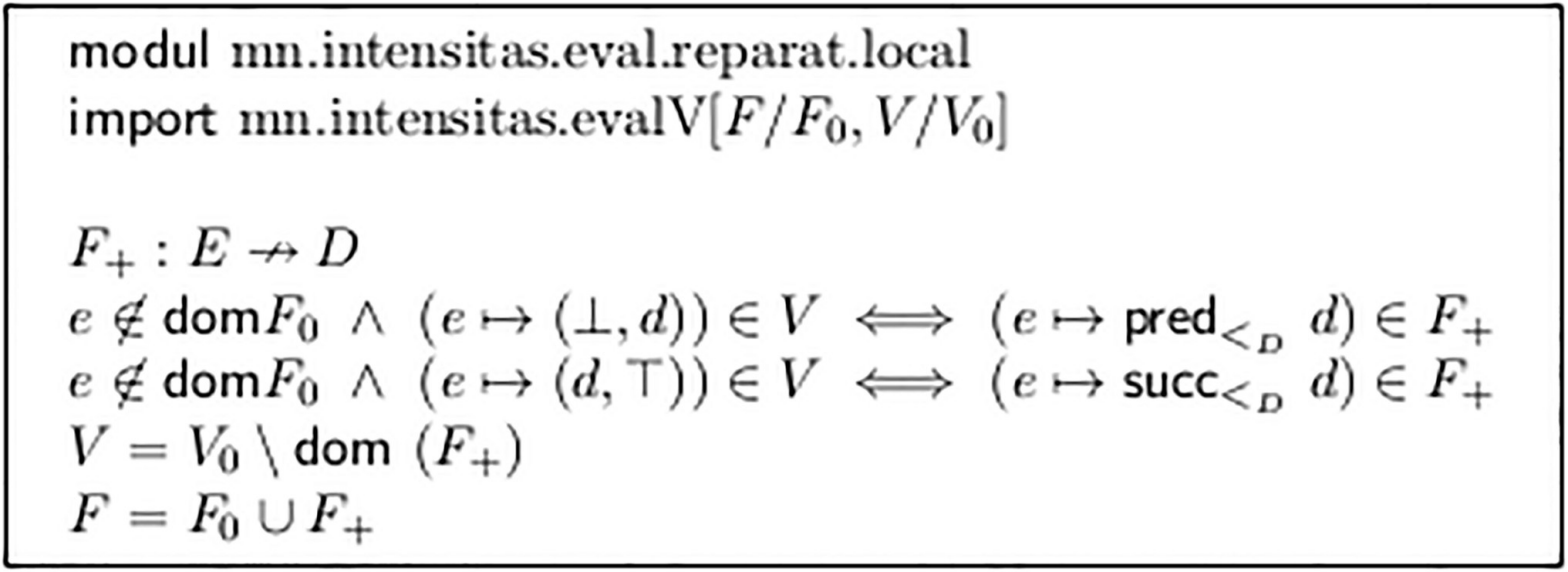
Local repair of partially undefined limits in *V*.

Module mn.intensitas.reparat.local in Fig 13 shows a typical technique: If defPart(V) holds, then each under-specified fork end is at least *partially* defined with a value *d* ∈ *D*. Now this entry is eliminated from *V* and instead the neighbor value from $\bar{D}$ (neighbor in the appropriate direction) is entered in *F*.

As far as we can judge from the outside view to the commercial music production frameworks, this seems to be there standard behavior: If, for example, in Sibelius a voice starts a crescendo fork at ***pp*** and does not define an end value immediately, then this is substituted as ***p***.

This technique seems to be a practical approach. It can be applied repeatedly: The extrapolated value is entered into *F* and thus possibly further constrains the end point of the next fork.

But it has several draw-backs: (a) It treats two adjacent forks in a different way from their join (see Lines h and i in Fig 8, in which the latter results from the former by applying *uniFurcas* from Fig 12.) This may be appropriate, but it is a significant property of the semantic interpretation step.

(b) This technique cannot be applied freely to fork ends from *V* with both lower and upper bounds. For example, in Fig 8 Line d, we can assign ***f*** to *e*_3_, but not ***ff*** to *e*_4_. Obviously in this case we need three value levels, not only two, between ***mf*** and ***ff***. This is accomplished by the algorithm in Section 2.10.

### 2.9 Alternative semantic models

The variants of semantic interpretation defined so far are just proposals to the domain experts, from which they can select those which are appropriate when considering all aspects of a work of art and its production contexts. Totally different alternatives are possible and indeed widely spread.

**Fig 14:**
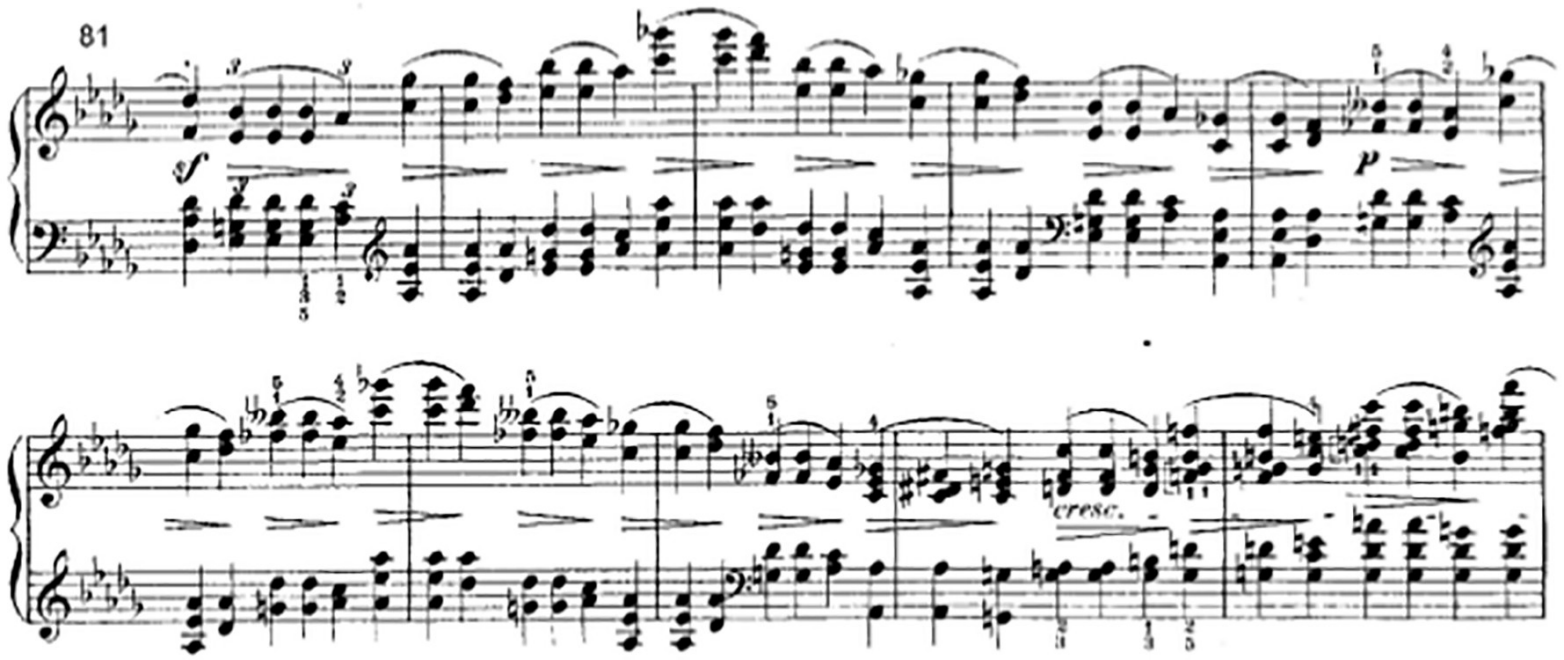
F. Chopin, Piano Sonata b flat minor op. 35, first movement: A different meaning of diminuendo symbols (edition [32], in the public domain).

**Fig 15:**
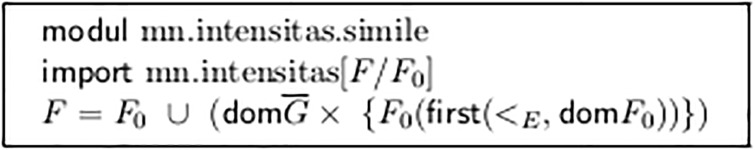
Alternative, very different semantics.

For example, Fig 14 shows the measures 81pp of the first movement of the b-flat-minor sonata by Chopin. [32] The effects of the diminuendo symbols are obviously not intended to accumulate, but instead to begin with the same dynamic level each. Thus, already our basic rule 7 from the initial Section 2.3 does not apply. This kind of usage is often applied to repeated similar motifs in some sub-sections of a piece and is thus called “mn.intensitas.simile” by us. But it may also apply to entire (short) compositions, as in “An den kleinen Radioapparat” by Hanns Eisler. Fig 15 shows the code which transforms this notation into our standard model, to make all analyses in this article applicable. If we apply this code to measures 85 (starting with the ***p***) to 89 (before the *cresc*.) from Fig 14, while modeling each chord as one event, then it adds a ***p*** at the start of each fork, which is obviously intended.

The same algorithm works for the beginning of the example only, if ***sf*** is treated as a “normal” dynamic value, which may cause other problems. Therefore in another edition the editors added a ***f*** to the second chord, thus *mn.intensitas.simile* is again applicable. [33]

Examples like these show clearly, that one single, normative and conclusive semantic interpretation system is neither sensible nor possible,—even in the narrow limits of one single and stylistic homogeneous composition: To decide the semantics of the forks, one most consider the motif identity and the overall dramatic curve. Nevertheless, once one particular semantic interpretation scheme has been selected, the further evaluation rules can and should be specified precisely.

### 2.10 Simple inter- and extrapolation by DDQ-analysis

**Fig 16:**
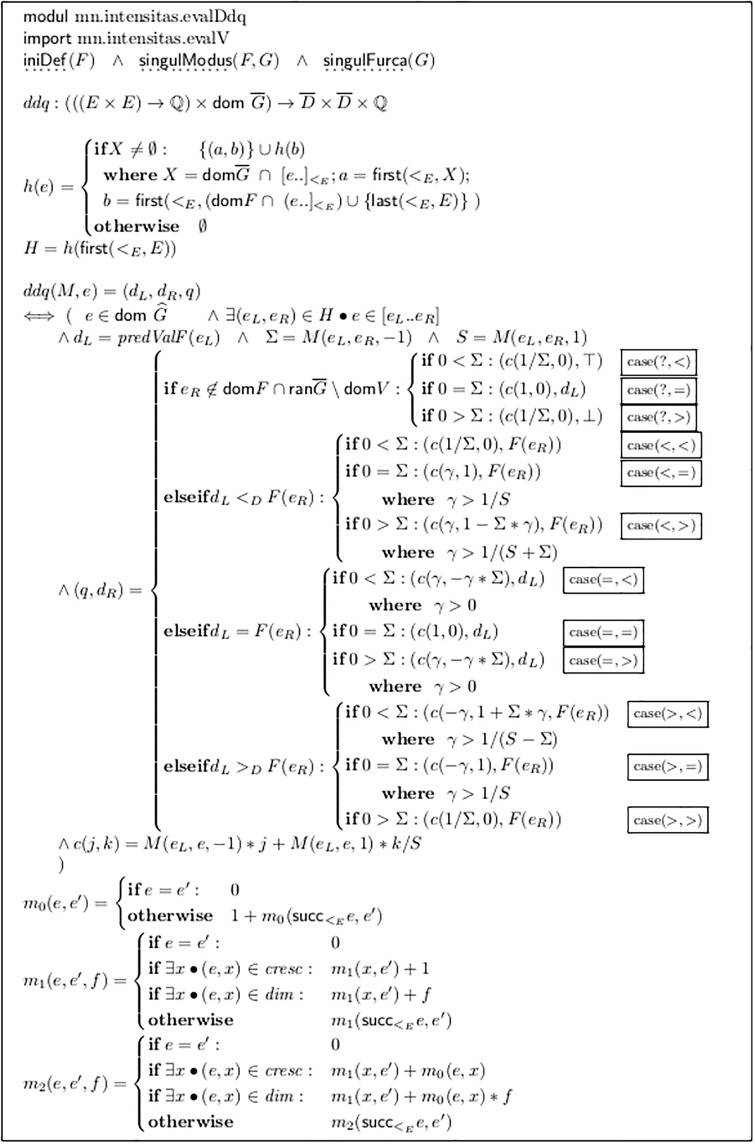
DDQ-analysis for inter- and extrapolating of fork groups.

Module mn.intensitas.evalDdq in Fig 16 shows an alternative to the above-mentioned local repair, namely a simple interpolation algorithm which also solves some cases of extrapolation, called DDQ-analysis. It processes maximal groups of adjacent forks which do not contain explicit dynamic values, but are framed by them. The idea is to find a natural interpretation for the contour constituted by the different lengths and directions of the forks.

The set *H* contains all these groups, represented by their limiting events. *H* is calculated with the auxiliary function *h*(*e*), which cuts off the leading group and applies itself recursively for the remaining events. (*X* are all events where a fork starts, at or after *e*; *a* is the first in this set; *b* is the first explicit dynamic value after *e*, or the very last event in *E*, as the terminating case.) There are four combinations of the definedness of the left and the right side of the group:

- The dynamic value at the left side of groups (*d*_*L*_) can only be undefined if ¬iniDef holds. As soon as the very first explicit dynamic appears in *F*, all subsequent fork groups always have a left side value, as dynamics are “sticky”, as defined by Number 6 of the informal description list in Section 2.3.
- On the right side, it is different, which again shows the asymmetric nature of music notation. The right side value of the group (*d*_*R*_) is undefined if and only if the next explicit dynamic value does not stand at the very end of the last fork but is separated by one or more events. In Fig 8 Line b group (*e*_2_, *e*_4_) and Line d group (*e*_2_, *e*_7_) have a defined endpoint, while Line c group (*e*_2_, *e*_5_) does not.

We claim iniDef and thus exclude groups with an undefined left value for the rest of this section.

The algorithm assigns to the end of each fork a value (*d*_*L*_, *d*_*R*_, *q*) of type $\bar{D}\times \bar{D}\times Q$. *d*_*L*_ and *d*_*R*_ are the framing dynamic values and *q* is a quotient indicating an intermediate position relative to these constants. For this, the algorithm applies a *measure* to the forks. The start of the very first fork in the group is aligned with event *e*_*L*_, and the end of the last fork is at event *e*_*R*_. The algorithm assigns to *e*_*L*_ the start value 0, and to each fork end the sum of all preceding measures in the group, divided by the measure over the whole group, Σ, from *e*_*L*_ to *e*_*R*_. The measure to apply is determined by the functional parameter *M*.

Some sensible measure definitions which can be used as *M* are defined directly in module mn.intensitas.evalDdq:

*m*_0_ is used as an ancillary function in *m*_2_ and simply counts events;
*m*_1_ assigns the constant value 1 to each fork, possibly negated for a diminuendo.
*m*_2_ assigns instead the number of spanned events, with signs as in *m*_1_.

Additionally, Section 2.11 below will define the measure *m*_*T*_ which considers the temporal durations of the forks. In all these functions, the additional parameter *f* can be set to −1 to measure diminuendo forks with negative values, thus yielding the overall crescendo distance as a result, or it can be set to + 1, measuring the length of all forks, independent of their orientation.

There are four cases of the framing explicit dynamics: *d*_*L*_ < *d*_*R*_, *d*_*L*_ = *d*_*R*_ and *d*_*L*_ > *d*_*R*_ and additionally the case that *d*_*R*_ is not known. These cases combine with three different results of applying the selected measure: 0 < Σ, 0 = Σ and 0 > Σ. We label these twelve cases with a 2-tuple of symbols {<, =, >, ?} × {<, =, >}.

Most natural are the cases (<, <) and (>, >), see Fig 16. For calculating the *q* values for the fork ends, the auxiliary function *c* is called with parameter *j* = 1/Σ, which normalizes all (oriented) measure results, so that the final value *q*(*e*_*R*_) = 1. (Only the first summand in the expression of the function definition contributes, because the second summand is set to *k* = 0—as will be discussed below).

**Fig 17:**
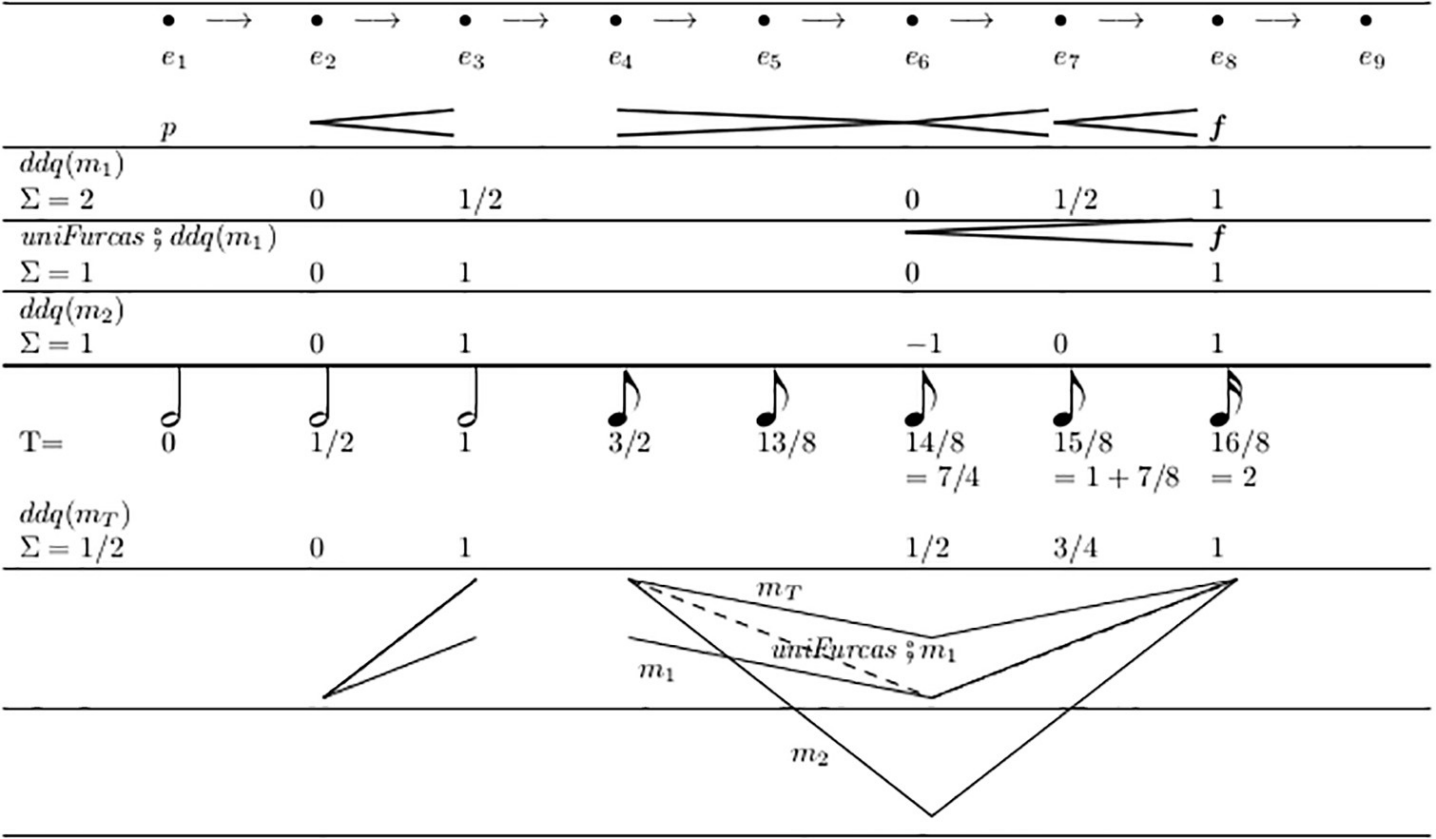
Examples of DDQ values by different measures, all in case (<, <).

Fig 17 shows an example: The vertical segments of the Figure show the resulting values for *q* when applying different measures; the curves in the bottom show the results graphically; *m*_*T*_ is explained in Section 2.11 below. Technically, DDQ is a free affine combination of *d*_*L*_ and *d*_*R*_. In a subsequent transformation step this must be mapped to some vector space, in which (*d*_*L*_, *d*_*R*_, *q*_*x*_) corresponds to the result of (1 − *q*) * *i*(*d*_*L*_) + *q* * *i*(*d*_*R*_), where “+” and “*” can be defined in various ways.

The DDQ value (*d*_*L*_, *d*_*R*_, 0) stands everywhere for the value of *i*(*d*_*L*_) and (*d*_*L*_, *d*_*R*_, 1) for *i*(*d*_*R*_). For all values 0 < *q*_*x*_ < 1 the resulting value lies between *i*(*d*_*L*_) and *i*(*d*_*R*_). Particularly, the appearance of a negative *q* value shows that the dynamic interpretation goes temporarily in the opposite direction of its overall tendency. Please note that the values for *q* can be somehow counter-intuitive: Due to the resulting linear projection (0, 1) ≡ (*d*_*L*_, *d*_*R*_), if *d*_*L*_ > *d*_*R*_, an increase in *q* means a decrease in dynamics.

In Fig 17, the second application of *m*_1_ is preceded by the application of *uniFurcas* from Fig 12. This unifies two adjacent forks into one: from *e*_6_ to *e*_8_. Choosing *ddq* and *m*_1_ as a semantic model implies that there is indeed a difference between notating one fork as a whole or split in two. This semantic difference will not appear with most other ways of interpretation.

It is an important gain that DDQ-analysis can even process totally unspecified fork ends with *V*(*e*) = (⊥, ⊤) (see the values calculated for *q* in line f of Fig 8). This is because considering also the distances on the horizontal axis adds a new quality of information, beyond the mere collection of limits as performed by V-analysis.

The case (=, =) is also a natural one as both tendencies (of framing dynamic values and fork contour) coincide. The above-mentioned normalization is not possible (division by zero), so *c* is called with *j* = 1. Nor would a normalization be of any significance: Being identical, the explicit dynamic values *d*_*L*_ and *d*_*R*_ define only the interpretation of *q* = 0. All other values of *q*, and the overall *ambitus*, must be defined by at least one additional and totally arbitrary definition, for example, by assigning a *d*_*X*_ > *d*_*L*_ to some *q* > 0, or a *d*_*X*_ < *d*_*L*_ to a *q* < 0. (This is done in the next step of the transformation pipeline; see Section 2.12.) Nevertheless, the sequence of *q* values indicates a sensible contour for dynamics, probably varying with the chosen measure.

A natural solution for the case (?, =) is to add *d*_*R*_ = *d*_*L*_, which also leads to the case (=, =).

The case (?, <) could be transformed into (<, <) by adding $d_{R}={succ}_{{<}_{D}}d_{L}$, as it is done by the primitive local repair mechanism (See Section 2.8). Instead, the algorithm in Fig 16 sets symbolically *d*_*R*_ = ⊤, meaning some still unspecified value but higher than *d*_*L*_. This allows us in the next interpretation step to take the *q* values into account when deciding how far the distance between *d*_*R*_ and *d*_*L*_ shall be. (In case of (***pp***, 〈0, 1/8, 7/8, 3/16, 1〉, ⊤) the right end could be set to ***f***, while with (***pp***, 〈0, 7, 1〉, ⊤) mapping it to ***p*** could be more appropriate).

Normalization of the *q* values is again applicable, so *q* = 0 stands for *d*_*L*_ and *q* = 1 stands for that still undefined end value.

Case (?, >) is treated analogously.

Thus in case (?, =) the end value can be completed in a natural way, but in all three cases (?, _) and in case (=, =) the ambitus is still totally unspecified.

Critical are the remaining *conflicting cases* where the tendencies differ, namely (<, >), (<, =), (>, <), (>, =), (=, <) and (=, >). They should only by applied if not avoidable by selecting an alternative measure.

**Fig 18:**
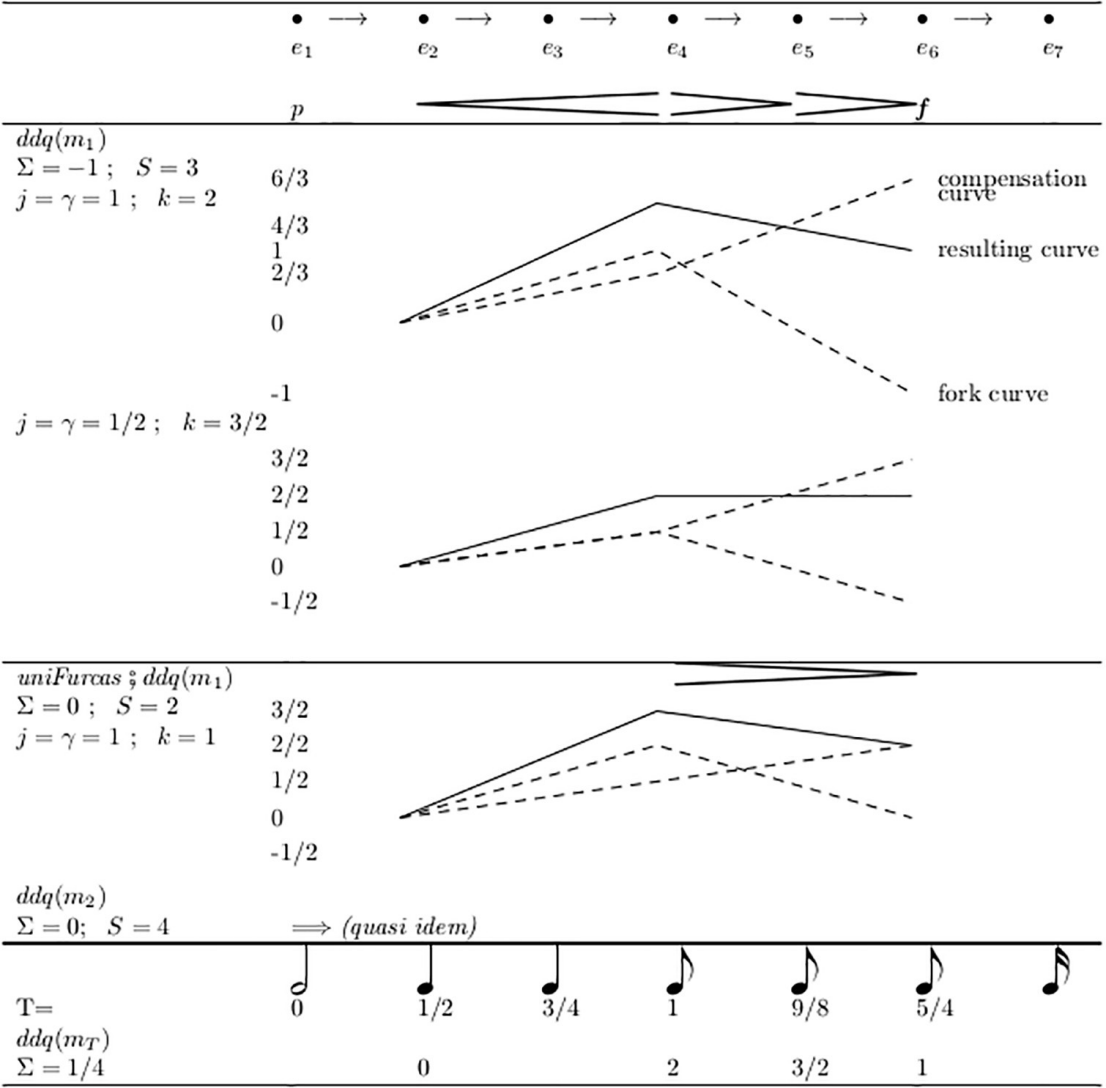
Less appropriate selections of measures, yielding cases (<, >) and (<, =).

Consider the case (<, >), where the framing dynamic values require a crescendo (e.g. from ***p*** to ***f***), while the measure delivers a curve with decreasing tendency and 0 > Σ (see Fig 18). Applying the same algorithm as above would of course set the final *q*(*e*_*R*_) = 1, because we divide by Σ, but since this Σ is negative, such a step would erroneously invert all fork directions. Instead, the final value for *q* must be corrected from Σ to + 1 by *adding* a second, positive and linear curve. This is realized by the second summand in function *c* and controlled by its argument *k*. For the very last position, *k* is completely added to the first term; for each intermediate position a certain fraction of *k* is added, interpolated in a linear way, by using the un-oriented version of the measure *M*, that is, by treating forks of both kinds identically. The overall sum is *S*, which is always positive, in contrast to Σ. This curve is called a *compensation curve* in the following.

The curve which is delivered by the first term of the function *c* and which represents the sequence of forks (and which has been used directly in all cases above) is called *fork curve*. It can be scaled by the argument *j*, which serves as a *calibration factor*. The sum of both curves is the *resulting curve*.

The first case in Fig 18 (using *m*_1_) shows an example: The fork curve is left unchanged (calibration factor *j* = 1), so it ends at −1. The compensation curve is controlled by *k* = 2. Both add to a resulting curve, ending at *q*(*e*_*R*_) = + 1, as required.

From the condition *q*(*e*_*R*_) = *c*(*e*_*R*_) = 1 follows immediately *k* = 1 − Σ*j. The parameter *γ* used for *j* must be >0 because the curve must not be inverted. Additionally, it is required that *j* > *k*/*S*, because otherwise the linear addition would *over-compensate* the diminuendos, as shown in the second triple of curves in Fig 18 with *j* = 1/2 and *k* = 3/2. Inserting the first condition into the second, we get *j* > (1 − Σ *j)/*S* ⇒ j*S > 1 − Σ *j ⇒ j*(*S* + Σ) > 1 ⇒ *j* > 1/(*S* + Σ). This is unsolvable for *S* = −Σ, which stands for all groups with only diminuendo forks. Of course, these cannot be corrected into an overall crescendo. But this case is excluded anyhow by V-analysis, which recognizes in $\begin{array}{ccc} p & \;\;>\;\; & f\\ e_{1} & & e_{2} \end{array}$ an “***f*** subito”, thus sets *V*(*e*_2_) = (***f***, ⊤) and leads to the case (?, >), due to the appearance of dom *V* in the first **if**… clause in Fig 16.

If the group contains at least one crescendo, *S* + Σ > 0 holds.

Candidates for “normal” interpretations are *c*(−1/Σ, 2), which normalizes the fork contour to end at −1, and *c*(1, 1 − Σ), which does not normalize, but selects the linear compensation accordingly. The parameter *γ* defines the relative weight between the fork curve and the compensation curve. The effects of different values of *γ* is an open question and possibly requires *empirical* research.

Case (>, <) has a fork curve with a positive Σ. But since the framing dynamic values do decrease, it must be inverted by using *j* = −*γ*, and shifted to *q*(*e*_*R*_) = + 1 by an appropriate *k*, as in the preceding case. (The inversion of all forks will be reverted by the final projection from 0 to + 1 into *e*_*L*_ > *e*_*R*_, as mentioned above.) The parameter *γ* is again positive, but the inverted Σ flows into its validity condition.

Case (<, =) is shown in the *M* = *m*_2_ case in Fig 18. Since Σ = 0, there is no natural notion of normalization, and each *γ* > 0 is a possible calibration factor. Since *e*_*L*_ < *e*_*R*_, the end point 0 must be corrected to 1, which is achieved by setting *k* = 1. This is constant and independent of *γ*. Nevertheless, *γ* again determines the relative weight between the fork contour and the linear compensation. Over-compensation is again avoided by *γ* > *k*/*S* = 1/*S*.

Case (>, =) is treated analogously: The curve is inverted by *j* = −*γ* and shifted by *k* = 1, again with *γ* > 1/*S*.

The case (=, <) means that the fork curve ends at some Σ > 0, but the resulting curve must end at *q* = 0, because the framing dynamic values are identical. Any calibration factor *j* = *γ* > 0 may by applied, and the end point is shifted to 0 by the linear compensation *k* = −*j* * Σ. Again, over-compensation is prevented by the condition *j* > *k*/*S* ⇒ *j* > (−*j* * Σ)/*S* ⇒ *j* * *S* > − *j* * Σ ⇒ *S* > −Σ, which is always fulfilled if the fork group contains at least one crescendo. This is again guaranteed by V-analysis, as described above.

The case (=, >) behaves exactly the same, but finally the compensation shifts *upward* towards 0 by the same formulas.

The last example in Fig 18 shows that the selection of *M* = *m*_*T*_ finally yields a Σ with the correct sign, so all cases above are indeed avoidable with this particular example.

### 2.11 Considering time values

**Fig 19:**
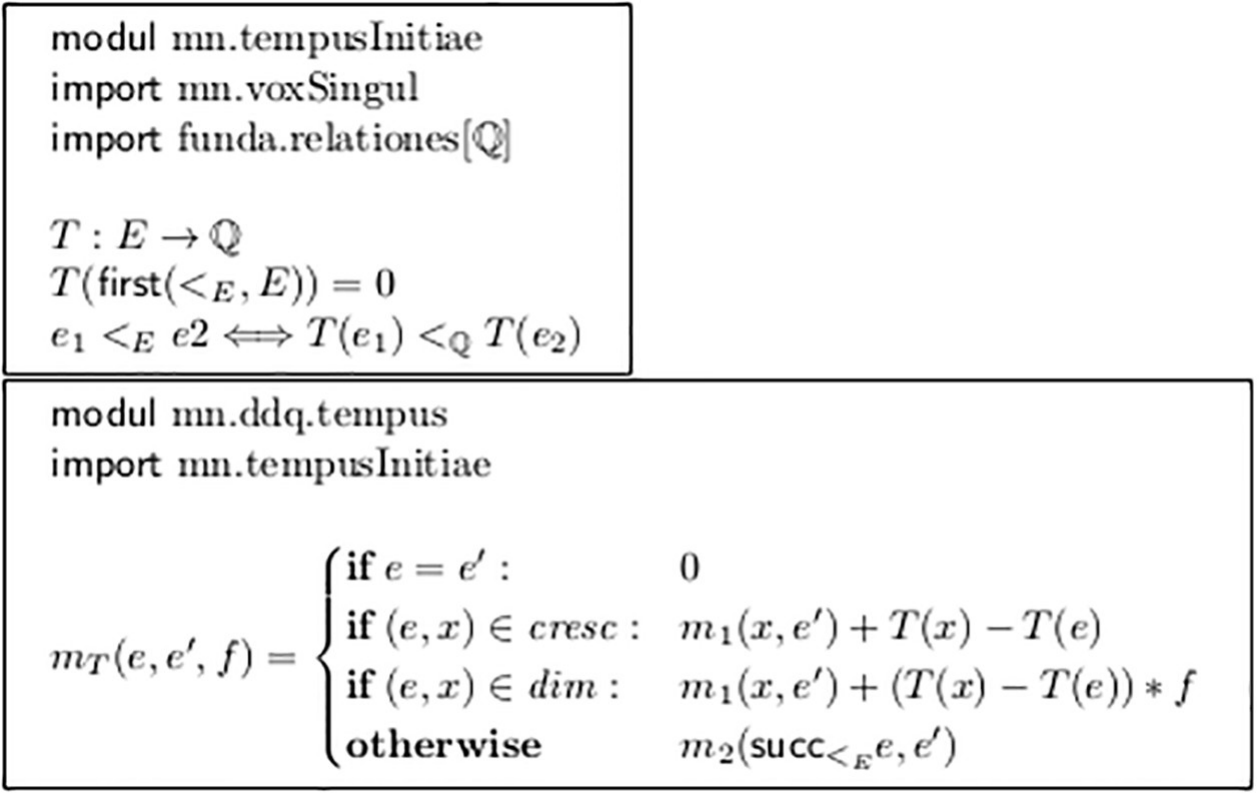
Considering events’ temporal start points.

Even with this very primitive semantic evaluation we now reach the border where the restriction to one single “musical parameter” can sensibly be given up. Module mn.tempusInitiae in Fig 19 introduces a *temporal parameter*, namely the start time point of the events, measured in rational numbers.

Module mn.ddq.tempus combines these with the ddq algorithm and defines the measure *m*_*T*_, which takes the *durations* of the forks, with signs as in *m*_1_. The final cases in Figs 17 and 18 illustrate the module’s application.

### 2.12 Further transformations

With progressing model construction, the number of variants and open decisions explodes. Up to now many points have remained open regarding the construction of the ddq values:

- The strategy of selecting the measure *M* can be
  - to find the least complex measure which leads to a non-contradictory case, separate for each fork group,
  - or to find one such measure, common for all fork groups,
  - or to find one for each of the three cases Σ < 0/ = 0/>0.
- Additional measures may be defined, such as counting events, but not below a particular metric level.
- An alternative algorithm may be designed which allows *different* measures for the fork curve and the compensation curve. (We assume that the effort to calculate the allowed ranges for the parameters will be dramatically greater).
- The different combinations of *M* and *γ* only *make proposals* of different interpretations to the user. Selecting among these will need further input. This input can come from
  - additional analysis of the notated data, such as in parallel parameter tracks,
  - or from general rules about *style and usus*,
  - or from a combination thereof.

The same holds of the further processing of the results:

- The results of DDQ-analysis are still *totally symbolic*. They are just a translation of the input, and whether they are to be interpreted as physiological, psychological or physical values, is still totally open.
- The results from different fork groups are possibly not comparable, especially if these represent different cases from {<, =, >, ?} × {<, =, >}.
- It cannot be derived how values like (***p***, ***f***, 1/2) and (***pp***, ***ff***, 1/2) are related. (But indeed it is likely that there exist “natural” interpretations, which have to be defined in the further processing steps explicitly).
- Even with identical *d*-values, the interpretation of the *q* is totally open. The curve between (***p***, ***f***, 0), (***p***, ***f***, 1/2) and (***p***, ***f***, 1) can be interpreted in a linear, logarithmic or exponential way, or even adjusted by an empirically given characteristic curve.

On the other hand, we have found some concrete results:

- Only the selection of the measure(s) flows in as an additional input. Beside this, DDQ-analysis only makes explicit the proportions contained in the fork contour implicitly.
- From the different measures and resulting cases, new properties can be defined which characterize pieces or groups of pieces, for example, for restricting the input to further processes.
- DDQ-analysis shows the fundamental difference between the cases and shows that some of them have the parameter *γ* as an additional degree of freedom. This can also be seen as an indicator for missing precision of the notation. So *γ* is possibly a concrete representation of the subconscious uneasiness, which drove composers of historic avant-garde to invent new and more precise notations for dynamics.
- But DDQ-analysis also calculates precise numeric limits for *γ*, which may not be exceeded without violating the intention of the notation. (This point and the preceding one are typical uncircumventables, as announced above).

## 3 Conclusion

In this paper we have looked upon musical notation systems as “precise languages” and applied methods well-proven for computer languages (see Section 2.1) for their specification. This led to a plethora of branching variants and collections of parameters and thus a unifying framework for their classification. Each of the defined notation systems can transform graphic input into a collection of data for further domain-oriented processing (see Figs 10 and 17).

**Fig 20:**
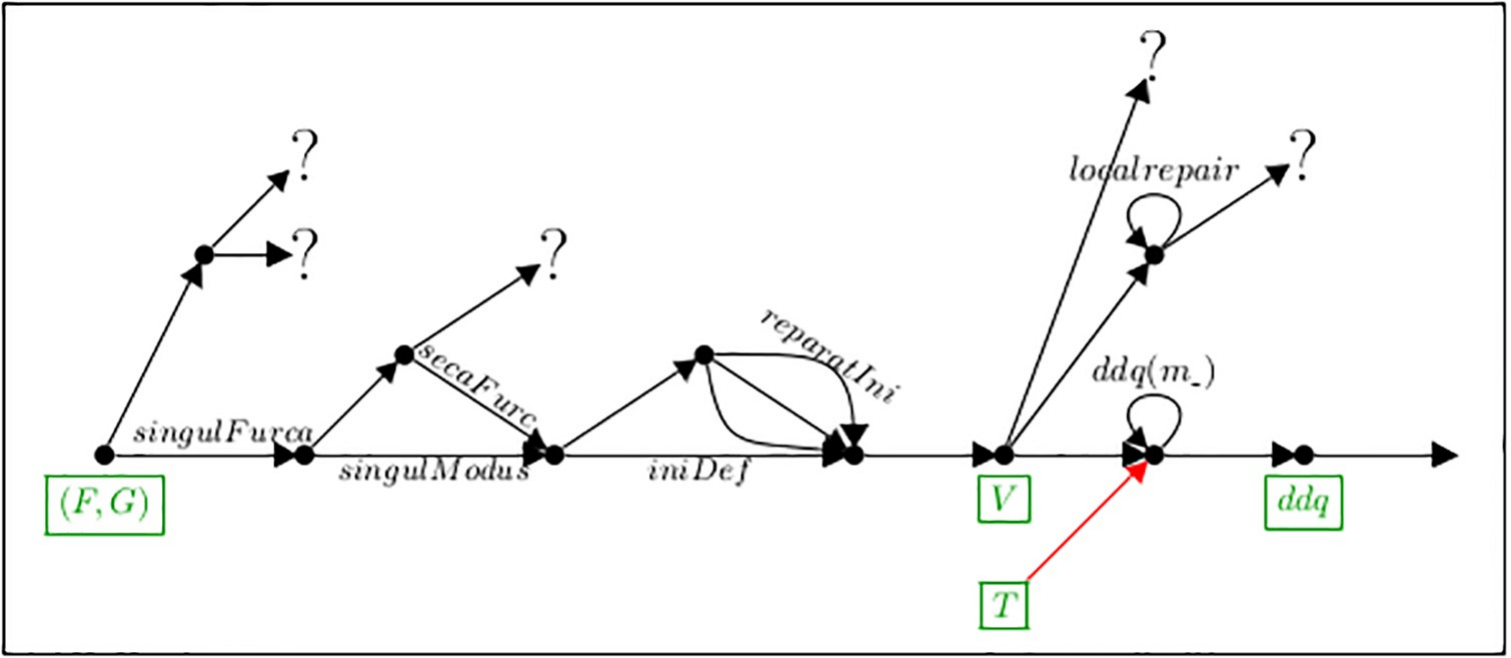
Described transformation paths.

Fig 20 shows the transformations and decisions described so far. One major role of maps like this is to allow the precise positioning of newly defined alternative semantics.

**Fig 21:**
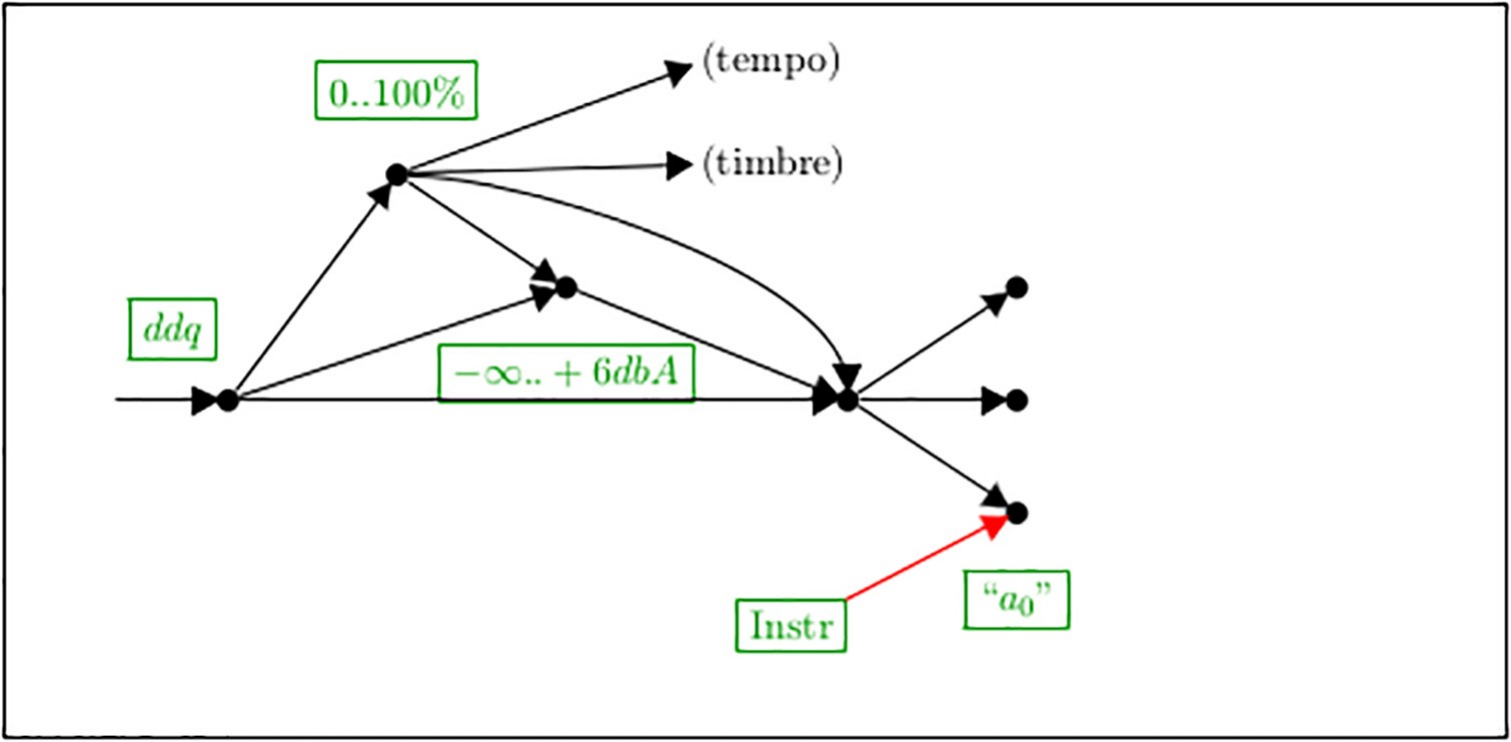
Possible further transformation paths.

Possible further translation steps and alternatives are shown in Fig 21: Following the general direction towards a final hypothetical automated execution, the ddq values must be further translated, for example, in relative volumes, and finally in physical parameters producing sound. These further processing steps naturally vary widely with style and usus. They form a network which first branches more and more and then runs together again at its end, for to realize it physically on a particular instrument.

For example, when aiming at digital sound synthesis, finally a parameter *a*_0_ must be calculated. Here the character of the executing instrument cannot be neglected any more, and it flows into the calculation chain as a further, independent parameter of the composition.

On the other hand, the notated information about dynamics is possibly not restricted to influence only the physical amplitude, but in most instrumental settings, it flows necessarily into parameters like timbre and duration. It may be translated additionally into tempo and agogic, depending on the epoch and interpretation style.

The numeric results of further transformation steps may not only be used for automated execution, but also be fed into further analyses and empirical evaluations, such as those executed in [17].

So, Fig 21 is in no way meant as complete, but as a map for orientation, waiting for all kinds of further processing steps to be entered.

Besides dynamics, our methodology can be applied analogously to any other aspect of music notation, yielding precise definitions, nomenclature and transformation networks. Selecting, interpreting and judging the nodes and parameters of these networks is left to the domain experts. But their selection is from now on explicit and documented.
